# Beyond glycemic control: the cardiac and hepatic benefits of SGLT2 and DPP-4 inhibitors in mitigating chronic cadmium-induced inflammation, oxidative/nitrative stress, apoptosis and fibrosis

**DOI:** 10.3389/fphys.2025.1752370

**Published:** 2026-01-21

**Authors:** Fatma E. Hassan, MennaAllah M. Hassanien, Asmaa Selmy, Lamiaa Mohamed Mahmoud, Amal Darwish, Basant A. Aldreny

**Affiliations:** 1 Medical Physiology Department, Kasr Alainy, Faculty of Medicine, Cairo University, Giza, Egypt; 2 Department of Physiology, General Medicine Practice Program, Batterjee Medical College, Jeddah, Saudi Arabia; 3 Anatomy and Embryology Department, Faculty of Medicine, Cairo University, Giza, Egypt; 4 Department of Medical Biochemistry and Molecular Biology, Faculty of Medicine, Ain Shams University, Cairo, Egypt

**Keywords:** cadmium, DPP-4i, heart, liver, SGLT2i

## Abstract

**Background:**

Cadmium (Cd) is a hazardous ecological contaminant implicated in substantial oxidative stress (OS), nitrative stress, inflammation, apoptosis and fibrosis, particularly in the heart and liver.

**Objective:**

This study aims to contrast the protective effects of “Canagliflozin; Cana” versus “Sitagliptin; Sita” in countering the chronic Cd-induced cardiac and hepatic damage.

**Methods:**

Four groups of adult male Wistar rats (6 each) were created: Control, Cd-exposed; rats received 100 mg/L CdCl_2_ via drinking water, Cd + Cana; rats received Cana 10 mg/kg, orally in parallel with CdCl_2_ (100 mg/L), and Cd + Sita; rats received Sita 10 mg/kg, orally concomitant with CdCl_2_ (100 mg/L). Following a 12-week course of treatment of all regimens, serum glucose, albumin, aspartate transaminase, alanine transaminase, lactate dehydrogenase, creatine Kinase-MB (CK-MB), Troponin I and Troponin C (cTnC) were measured. Cardiac and hepatic tissues were subjected to quantitative real-time polymerase chain reaction assays for Notch1, transforming growth factor-β, SMAD3, alpha-smooth muscle actin (α-SMA), and SMAD7 expression levels. In addition, interleukin-10/-1β, tumor necrosis factor-α, reduced glutathione, and malondialdehyde were evaluated. Besides, there was a cardiac and hepatic histological evaluation after hematoxylin and eosin, and Masson staining, as well as immunohistochemistry measurement of caspase 3, nuclear factor kappa B (NF-κB) and inducible nitric oxide synthase (iNOS). Results were analyzed using one-way ANOVA followed by Tukey’s *post hoc* and then represented as mean ± standard deviation. Differences among groups were considered statistically significant when p value is ≤0.05.

**Results:**

There were no substantial changes in blood glucose levels across all groups, which confirmed the model’s normoglycemic nature. Therefore, independent from their glycemic effect, both Cana and Sita significantly but comparably (p > 0.05) improved cardiac and hepatic OS, inflammation, apoptosis, and fibrosis associated with chronic Cd exposure. However, Cana demonstrated greater improvement (p < 0.05) in serum CK-MB and cTnC, cardiac (α-SMA) and hepatic (collagen area%) fibrosis, cardiac and hepatic apoptosis (caspase 3%), inflammation (NF-κB%) and nitrative stress (iNOS%) and restored their architecture.

**Conclusion:**

Both medications showed comparable cardio-hepatic protective effects. Yet, Cana outperformed Sita as a potentially effective therapy to counteract the negative consequences of chronic Cd-induced cardiac and hepatic pathologies.

## Graphical Abstract

**Figure:**
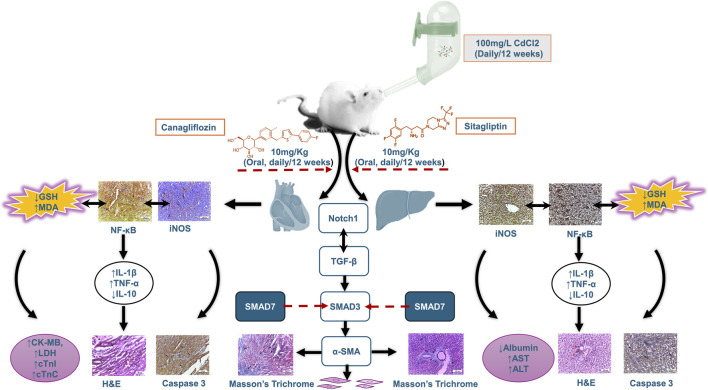

## 1 Introduction

Cadmium (Cd) is a serious non-biodegradable environmental contaminant. It can build up considerably as a result of occupations like iron extraction and mining. In addition to being a principal toxin in tobacco products. Among the primary ways that Cd is absorbed is consuming polluted food or water, as well as through breathing in ambient air (Li H. et al., 2019). Cd is a potent multi-organ toxin. Since its biologic half-life exceeds more than 20 years, it can accumulate and destroy many organs (Bhattacharjee et al., 2020). While its nephrotoxicity is well-established (Turkington et al., 2025), the mechanisms by which it induces synchronous chronic cardiotoxicity and hepatotoxicity still merits further exploration.

Because exposure to Cd disrupts the antioxidative defense mechanisms, reactive oxygen species (ROS) emerge, triggering inflammatory and apoptotic reactions, which may contribute to hepatotoxicity and cardiovascular disorders (Everett and Frithsen, 2008; Peters et al., 2010; Khan et al., 2022). Mortality from heart disease was shown to be strongly correlated with liver Cd levels (Voors and Shuman, 1977; Tellez-Plaza et al., 2012). Cd impairs the cardiac angiogenesis causing subsequent cellular hypoxia, damage or necrosis (Dong et al., 2009). Afterwards, myocardial cells trigger an inflammatory reaction, leading to myocardial fibrosis and ultimately heart failure (Frangogiannis, 2021).

Progression from inflammation to fibrosis causes permanent organ damage. In support, previous research reported that prolonged exposure to Cd increases the probability for cardiac (Türkcan et al., 2015) and hepatic (Hu and Phan, 2016) fibrosis. The hallmarks of fibrosis are surplus connective tissue growth and extracellular matrix (ECM) accumulation, which gradually impair the tissues’ characteristics resulting in malfunction (Bakalenko et al., 2024). Transforming growth factor-β (TGF-β) is crucial for fibrosis in any tissue, involving heart and liver (Xu et al., 2016; Kang et al., 2025). Alike, Notch signaling governs the myofibroblast activation in the liver and other organs (Hu and Phan, 2016; Xu et al., 2016; Wasnick et al., 2023; Wang et al., 2024). Notch activation induces cardiac mesenchymal cells’ alpha-smooth muscle actin (α-SMA) expression (Docshin et al., 2023). Likewise in liver fibrosis, Notch and TGF-β are positively cross-regulated (Mancarella et al., 2022). Notch1 stimulates TGF-β1 synthesis and SMAD3 phosphorylation, which increases α-SMA expression and promotes fibrosis (Aoyagi-Ikeda et al., 2011). Most literature indicates that the heart’s Notch signaling inhibits fibrosis (Zhou et al., 2019; Zhang et al., 2022). However, others suggested that Notch signaling has a profibrotic impacts and identified a synergistic connection between TGF-β and Notch in cardiac fibrosis (Russell et al., 2011; Li et al., 2021).

Despite the proven toxicity of Cd, there is a substantial knowledge deficit concerning the molecular pathways driving chronic Cd-induced cardiac and hepatic fibrosis, specifically the potential implication of the Notch1/TGF-β1/SMAD3/7 signaling axis. In addition, there is no solid preventive approach for chronic Cd induced tissues’ toxicity (Türkcan et al., 2015). Thus, research is desperately required, for finding pharmacological regimens to target this pathway which can prevent or mitigate chronic organ damage caused by prolonged Cd exposure.

Canagliflozin (Cana), dapagliflozin and empagliflozin - being sodium-glucose cotransporter-2 inhibitors (SGLT-2is)- serve as anti-hyperglycemic medications by reducing blood glucose levels in proximal renal tubules. Other pluralistic benefits of SGLT-2is, however, include lowering blood pressure, reducing inflammation, and protecting the cardiovascular system (Al Hamed and Elewa, 2020). In particular, Cana is particularly effective against liver (Hassan et al., 2024; Kalantari et al., 2024) and heart (Hasan et al., 2020a; Dasari et al., 2023) diseases.

Similarly, Sitagliptin “Sita,” a selective dipeptidyl peptidase-4 inhibitor (DPP-4i), displays a notable effectiveness in the treatment for those with diabetes. Sita, nevertheless, has shown a number of advantageous effects on animals with normoglycemia without causing hypoglycemia (Arab et al., 2021). Gliptins could mitigate the oxidative stress (OS), inflammation, and apoptosis along with their antifibrotic properties on liver (Wang X. et al., 2018) and heart (Esposito et al., 2017).

Whereas both SGLT2is, such as Cana, and DPP-4is, such as Sita, have demonstrated multifaceted “off-target” anti-inflammatory and anti-fibrotic attributes in diabetic animals, their comparative effects in a non-diabetic, chronic toxicological circumstances have not been studied. This necessitates the importance to figure out the most effective treatment classes for alleviating multi-organ injury from environmental contaminants like Cd, perhaps enabling an emerging clinical indication for these drugs “beyond glycemic control”.

Since Cd accumulates in tissues over decades, discovering pharmaceutical regimens that may be delivered in high-risk settings to avoid chronic organ damage is an urgent public health issue. Thus, this work employs a concurrent exposure-treatment model to simulate such preventive interventions. We investigated whether early administration of SGLT2 or DPP-4 inhibitors can intercept the inflammatory and fibrotic cascades triggered by chronic Cd exposure. Therefore, this research aims to evaluate the comparative protective potential of Cana and Sita against chronic Cd-induced cardiac and hepatic insult to elaborate which class provides more favorable tissue-specific protection. We investigated their antioxidant, anti-inflammatory, antiapoptotic and antifibrotic properties, which may offer a comprehensive prophylaxis or remedy to the health issues caused by this non-biodegradable contaminant.

## 2 Materials and methods

### 2.1 Declaration of animal use and ethical approval

This study was rigorously complying with the ARRIVE guidelines and was carried out in accordance with the U.K. Animals (Scientific Procedures) Act, 1986 and associated guidelines, EU Directive 2010/63/EU for animal experiments and the National Institutes of Health guide for the care and use of Laboratory animals (NIH Publications No. 8023, revised 1978. Further, the Cairo University (CU) Institutional Animal Care and Use Committee (IACUC) in Egypt approved this experiment: Approval number (CU III F 7 25).

### 2.2 Drugs and chemicals

Sigma-Aldrich (St. Louis, MO, United States) supplied the cadmium chloride (CdCl2 × 2.5 H2O), Janssen Pharmaceutical Co. (Titusville, NJ, United States) provided the canagliflozin, and Adamas Reagent Co. (Shanghai, China) provided the sitagliptin. Except otherwise specified, all kits were acquired from My BioSource, Inc. in San Deigo, United States and antibodies from Abcam, Cambridge, United Kingdom.

### 2.3 Animals’ grouping

Twenty-four mature male Wistar rats weighing 220 ± 7 g, acquired from Faculty of Medicine’s animal house, CU, Egypt, were kept in well-ventilated plastic cages (three per cage) at 25 °C ± 5 °C with regular light/dark rhythm for 7 days. Everyday rats were provided with *ad libitum* access to a standard chow diet**,** along with unlimited availability of water. Computer-generated randomization technique was utilized to distribute the rats evenly among the subsequent groups: Control (CTRL): rats received 0.9% saline by oral gavage (Kalantari et al., 2024). Cadmium (Cd): Rats received 100 mg/L CdCl_2_ via drinking water; a dose previously shown to effectively induce chronic multi-organ damage in Wistar rats (Türkcan et al., 2015). Cadmium + Cana (Cd + Cana): Rats received Cana 10 mg/kg, orally. This dose was selected based on its reported anti-inflammatory and anti-fibrotic efficacy in rat models of organ injury without inducing hypoglycemic adverse effects, ensuring the biological relevance of the findings to non-diabetic multi-organ protection (Kalantari et al., 2024; Bishr et al., 2025) in parallel with CdCl_2_ (100 mg/L). Cadmium + Sita (Cd + Sita): Rats received Sita 10 mg/kg, orally-this dose was chosen for its ability to provide “off-target” protective benefits in non-diabetic rats without affecting basal glucose levels (Arab et al., 2021)- concomitant with CdCl_2_ (100 mg/L). The pharmacological regimens were administered once daily concurrently with CdCl2 from the beginning of the 12-week exposure period to model a prophylactic intervention strategy, aimed at preventing the establishment of chronic multi-organ pathologies.

Blood samples were obtained 24 h after the experimental protocol concluded, then rats underwent cervical dislocation after administering ketamine/xylazine (60/6 mg/kg) cocktail (Hassan et al., 2025). The heart and liver were dissected, separated into two equal-sized sections, and handled accordingly for further examination.

### 2.4 Measured parameters

To ensure objectivity and eliminate bias, all biochemical, molecular and histopathological analyses were conducted blindly without respect for experimental group allocation.

#### 2.4.1 Blood parameters

The subsequent blood parameters were evaluated utilizing ELISA kits in compliance with manufacturer’s (My BioSource, Inc. in San Deigo, United States) directives: Glucose (Cat# MBS7233226), Albumin (Cat# MBS1600276), Aspartate transaminase (AST) (Cat# MBS264975), Alanine transaminase (ALT) (Cat# MBS269614), Interleukin (IL)-10 (Cat# MBS355232), IL-1β (Cat# MBS355368), Tumor necrosis factor-α; TNF-α (Cat# MBS2507393), Creatine kinase-MB (CK-MB) (Cat# MBS2515061), Lactate dehydrogenase (LDH) (Cat# MBS269777), Troponin I (cTnI) (Cat# MBS727624), and Troponin C (cTnC) (Cat# MBS7255103).

#### 2.4.2 Cardiac and hepatic tissues parameters

##### 2.4.2.1 OS markers

According to manufacturer’s instructions (My BioSource, Inc. in San Deigo, United States), the subsequent OS indicators malondialdehyde (MDA) (Cat# MBS738685) and reduced glutathione (GSH) (Cat# MBS724319) were assessed in hepatic and cardiac tissues with competitive and sandwich ELISA kits, respectively.

To verify the biological significance and comparability of cardiac and hepatic tissues biochemical parameters MDA, GSH, Il-1β, TNF-α and IL-10, all results were standardized to the total protein content of the relevant tissue homogenate of each sample which was determined by the Bradford assay. Then, the OS and inflammatory biomarkers were expressed in µmol/mg protin and pg/mg protein, respectively.

##### 2.4.2.2 Quantitative reverse transcription-polymerase chain reaction (qRT-PCR) of cardiac and hepatic tissues Notch1, TGF-β, SMAD3/7, and α-SMA mRNA relative expression

The TRIzol reagent (Invitrogen, Waltham, MA, United States) was employed for obtaining RNA from 30 mg of liver and heart tissues. A nucleic acid spectrophotometer (Thermo Fisher Scientific, Waltham, MA, United States) was applied to quantify the RNA amount. RNA (100 ng) was utilized to reverse-synthesize cDNA via Transcriptor First Stand cDNA Synthesis Kit. After that, DNA was amplified utilizing SYBR Green PCR Master Mix kit under the necessary reaction circumstances: 25 °C (10 min (min)), 50 °C (60 min) and 85 °C (5 min). To guarantee high-quality quantitative data, all samples and standard curves have been investigated in technical triplicate. The amplification efficiency of each primer pair was assessed by creating a 5-point standard curve from successive dilutions of pooled cDNA; all analyses revealed efficiencies ranging from 94% to 105%, with correlation coefficients (*R* ^2^) more than 0.99. The internal reference was GAPDH. Its utility as a normalizer was demonstrated by its consistent expression stability among all groups, with no significant fluctuation in threshold cycle (*C*_*t*_) values seen in spite of exposure to Cd (p > 0.05). Using the 2^−△△CT^ formula, the relative fold changes in mRNA expression of Notch1, TGF-β, SMAD3, α-SMA, and SMAD7 were determined. The following were the primer sequences (Shanghai Bioengineering Co., Ltd.): Notch1: Forward (F); TCGTGCTCCTGTTCTTTGTG, reverse (R); TCTCTCCGCTTCTTCTTGC, TGF-β: F; ATTCCTGGCGTTACCTTGG, R; AGCCCTGTATTCCGTCTCCT, SMAD3: F; AACTGCAGTGCCGCTATCC, R; CCAGCGGGGAAGTTAGTGTT, α-SMA: F; CTGAGCGTGGCTATTCCTTC, R; CGTCATACTCCTGTTTGCTGA, SMAD7: F; GTGGCATACTGGGAGGAGAA, R; TTGTTGTCCGAATTGAGCTG, and GAPDH (internal control): F; ATGACTCTACCCACGGCAAG, R; CTGGAAGATGGTGATGGGTT.

#### 2.4.3 Histopathological parameters

##### 2.4.3.1 Hematoxylin and eosin (H&E) and Masson’s Trichrome (MT) stain

Heart and liver tissues were dehydrated in increasing ethanol grades after being embedded in a 10% neutral buffered formalin solution (2 days). Then cleaned with xylol, and submerged into blocks of molten paraffin wax at 37 °C. The prepared paraffin blocks were sliced (thickness of 5 µm) and stained with H&E to examine histological features and MT stain to identify collagen fibers (Bancroft and Layton, 2018).

##### 2.4.3.2 Immunohistochemical (IHC) staining

A 1: 1000 dilution of the rabbit polyclonal antibody (ab2302) was utilized. The positive caspase 3 reaction manifested as brown cytoplasmic staining. A 1:20 dilution of a rabbit monoclonal antibody against nuclear factor kappa B; NF-κB p65 (ab86299) was employed. The positive NF-κB reaction exhibited as brown nuclear/perinuclear staining. 1:200 dilution of a rabbit polyclonal antibody against inducible nitric oxide synthase; iNOS (ab15323) was used. Brown cytoplasmic staining was a sign of an iNOS-positive reaction. To extract antigens, sections for IHC stains were heated in citrate buffer (10 min; pH 6.0). Nonspecific endogenous peroxidase activity was inhibited by applying a hydrogen peroxide solution. To prevent nonspecific binding, the sections were first rinsed with phosphate buffer saline (PBS) for 15 min, and then 10% normal serum was added for 30 min. The sections were subsequently rinsed for an hour in PBS with a diluted primary antibody. After rinsing, a biotinylated goat secondary antibody was added, and then streptavidin-peroxidase was incubated over 30 min. Using the hematoxylin “counterstain” and 3, 3-diaminobenzidine (DAB) “chromogen,” the coloration was accomplished (Kiernan, 2015).

##### 2.4.3.3 Histomorphometry studies

Leica Qwin 500 image analyzer computer system was used for quantitative analysis. The program’s measurement units (pixels) were automatically converted to actual measurement units (μm) by the image analyzer to ensure accuracy across groups. Six separate microscopic zones were selected at random for each section at a magnification of ×200 by a blinded investigator. The collagen area % in sections dyed with MT stain was measured. In IHC stained sections, the caspase 3, NF-κB and iNOS area % of positive immunoreactions were also assessed.

### 2.5 Statistical analysis

Data was handled using SPSS 26. Shapiro-Wilk test was used to ensure the normal distribution of data. One-way Analysis of Variance (ANOVA) was used to assess differences among all groups, accompanied by Tukey’s *post hoc* test for multiple comparisons. Afterwards, the results were displayed as mean ± standard deviation. p ≤ 0.05 reveals significance. Exact p-values were reported unless p < 0.001 (it was reported as (p < 0.001). Additionally, 95% confidence intervals (CI) were calculated for key biochemical and molecular parameters to indicate the precision of our estimates.

## 3 Results

### 3.1 Blood biochemical results

As displayed in Table 1, no significant effect on blood glucose was observed in any grp ensuring the normoglycemic nature of the experimental model and indicating that any protective effect in treated groups is glucose independent. The blood ALT, AST, CK-MB, LDH, cTnI and cTnC significantly increased in Cd grp vs. CTRL with a significant drop in albumin concentration. On contrary, all these parameters significantly improved in both treated groups (Cd + Cana and Cd + Sita) relative to Cd grp values. On every metric, Cana and Sita’s impacts were similar except for CK-MB and cTnC, which improved more in Cd + Cana grp. Furthermore, in contrast to CTRL grp, both ALT and AST completely normalized in Cd + Cana while only AST completely recovered in Cd + Sita grp.

**TABLE 1:** Blood albumin, ALT, AST, CK-MB, LDH, cTnI and cTnC results.

Parameter	CTRL	Cd	Cd + Cana	Cd + Sita
	Mean ± SD [95%CI]			
Glucose mg/dL	109.00 ± 13.48 [94.9, 123.1]	113.50 ± 10.45 [102.5, 124.5]	111.83 ± 9.02 [102.4, 121.3]	116.67 ± 8.36 [107.9, 125.4]
Albumin (g/dL)	3.96 ± 0.36 [3.58, 4.33]	2.16 ± 0.18^*^ [1.97, 2.34] ^*^p < 0.001	3.18 ± 0.10^*$^ [3.08, 3.29] ^*$^ p < 0.001	3.03 ± 0.15^*$^ [2.87, 3.19] ^*$^ p < 0.001
Alanine transaminase (U/mL)	33.80 ± 2.72 [31.0, 36.7]	60.18 ± 5.90^*^ [54.0, 66.4] ^*^p < 0.001	38.49 ± 3.39^$^ [34.9, 42.0] ^$^ p < 0.001	43.66 ± 2.45^*$^ [41.1, 46.2] ^*^p = 0.001 ^$^ p < 0.001
Aspartate transaminase (U/mL)	35.98 ± 2.82 [33.0, 38.9]	65.02 ± 8.55^*^ [56.1, 74.0] ^*^p < 0.001	42.43 ± 4.91^$^ [37.3, 47.6] ^$^ p < 0.001	42.56 ± 6.53^$^ [35.7, 49.4] ^$^ p < 0.001
Creatine Kinase-MB (U/L)	7.87 ± 0.95 [6.9, 8.9]	37.06 ± 3.34^*^ [33.6, 40.6] ^*^p < 0.001	16.36 ± 1.51^*$^ [14.8, 17.9] ^*$^ p < 0.001	20.78 ± 1.42^*$#^ [19.3, 22.3] ^*$^ p < 0.001 ^#^p = 0.006
Lactate dehydrogenase (U/L)	111.87 ± 10.49 [100.9, 122.9]	215.05 ± 8.48^*^ [206.2, 224.0] ^*^p < 0.001	157.60 ± 13.74^*$^ [143.2, 172.0] ^*$^ p < 0.001	174.32 ± 15.36^*$^ [158.2, 190.4] ^*$^ p < 0.001
Troponin I (ng/mL)	0.04 ± 0.04 [0.0, 0.09]	0.27 ± 0.03^*^ [0.23, 0.3] ^*^p < 0.001	0.17 ± 0.04^*$^ [0.13, 0.21] ^*^p < 0.001 ^$^p = 0.006	0.19 ± 0.001^*$^ [0.18, 0.21] ^*$^ p < 0.001
Troponin C (ng/mL)	0.14 ± 0.04 [0.1, 0.17]	0.68 ± 0.10^*^ [0.57, 0.78] ^*^p < 0.001	0.30 ± 0.07^*$^ [0.23, 0.37] ^*^p = 0.002 ^$^ p < 0.001	0.43 ± 0.04^*$#^ [0.39, 0.47] ^*$^ p < 0.001 ^#^p = 0.013

Data was depicted in means ± standard deviations.*p* ≤ 0.05 is significant. *: Significant vs. CTRL, $: Significant vs. Cd, #: Significant vs. Cd+Cana. CTRL: control, Cd: Cadmium, Cana: Canagliflozin, Sita: Sitagliptin,95% CI (95% Confidence Interval).

### 3.2 Cardiac and hepatic OS biochemical results

**FIGURE 1:**
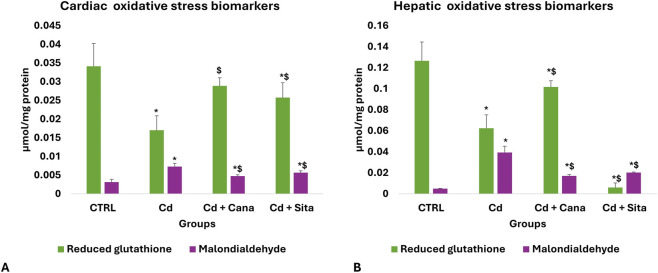
Cardiac **(A)** and hepatic **(B)** oxidative stress biomarkers. Data was displayed in means± standard deviations (n = 6). Statistical significance was determined by one-way ANOVA followed by Tukey’s *post hoc* test. *p* ≤ 0.05 is significant. *: Significant vs. CTRL, $: Significant vs. Cd. CTRL: Control, Cd: Cadmium, Cana: Canagliflozin, Sita: Sitagliptin.

As shown in Figure 1, Cd grp’s GSH significantly decreased concomitantly with a substantial MDA increase (p < 0.001) in cardiac and hepatic tissues vs. CTRL. In contrast to Cd grp, there was a significant improvement in the Cd + Cana grp’s GSH and consequently MDA in the cardiac (p = 0.001 and <0.001, correspondingly) and hepatic (p < 0.001) tissues attaining a comparable beneficial effect to that of Sita on the cardiac (p = 0.010 and 0.001, correspondingly) and hepatic (p = 0.028 and <0.001, correspondingly) tissues of the Cd + Sita grp. However, the cardiac MDA in Cd + Cana (p = 0.002), and GSH and MDA in the Cd + Sita (p < 0.001) grp as well as the hepatic GSH and MDA in the Cd + Cana (p = 0.019 and <0.001, respectively) and Cd + Sita (p < 0.001) did not attain the CTRL values. Of note, the cardiac GSH totally normalized (p > 0.05) in the Cd + Cana while there was an existing significance in the cardiac GSH of the Cd + Sita grp (p = 0.019) when compared to CTRL values. Despite the antioxidant effects of Cana were superior to those of Sita, no statistical significance was recognized between both therapeutics.

### 3.3 Cardiac and hepatic inflammatory biochemical results

**FIGURE 2:**
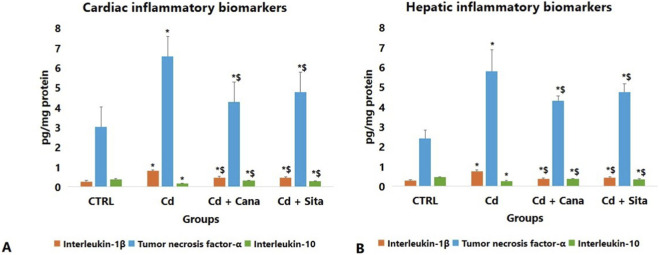
Cardiac **(A)** and hepatic **(B)** inflammatory biomarkers. Data was displayed in means± standard deviations (n = 6). Statistical significance was determined by one-way ANOVA followed by Tukey’s *post hoc* test. *p* ≤ 0.05 is significant. *: Significant vs. CTRL, $: Significant vs. Cd, CTRL: Control, Cd: Cadmium, Cana: Canagliflozin, Sita: Sitagliptin.

As presented in Figure 2, IL-1β and TNF-α significantly increased concomitantly with a substantial decline in IL-10 in cardiac (p < 0.001, 0.003 and <0.001, respectively) and hepatic (p < 0.001) tissues of Cd grp vs. CTRL. In contrast to Cd grp, there was a significant improvement in IL-1β, TNF-α and IL-10 in cardiac (p < 0.001) and hepatic (p < 0.001, 0.002 and <0.001, respectively) tissues of the Cd + Cana attaining a comparable beneficial effect like that of the cardiac (p < 0.001) and hepatic (p < 0.001, 0.037 and 0.001, respectively) tissues of the Cd + Sita grp. However, the cardiac IL-1β, TNF-α and IL-10 in Cd + Cana (p < 0.001, 0.003 and <0.001, respectively) and Cd + Sita (p < 0.001) grp as well as the hepatic IL-1β, TNF-α and IL-10 in Cd + Cana (p = 0.035, <0.001 and 0.004, correspondingly) and Cd + Sita (p = 0.001, <0.001and <0.001, respectively) groups did not attain standards when compared to CTRL grp. Despite the anti-inflammatory effects of Cana were superior to those of Sita, no significance was detected between both therapeutics.

### 3.4 Cardiac and hepatic molecular results

**FIGURE 3:**
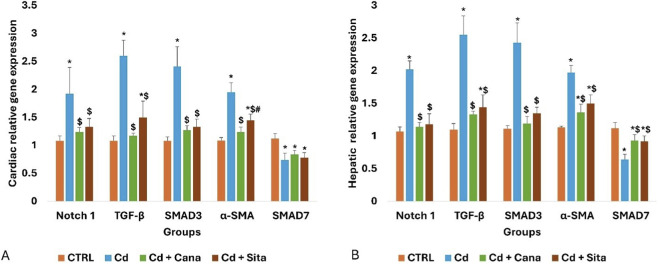
Cardiac **(A)** and hepatic **(B)** relative expression of Notch1, TGF-β, SMAD3, α-SMA and SMAD7. Data was exhibited as mean± standard deviation. (n = 6). Statistical significance was determined by one-way ANOVA followed by Tukey’s *post hoc* test. *p* ≤ 0.05 is significant. *: Significant vs. CTRL, $: Significant vs. Cd, #: Significant vs. Cd+Cana. CTRL: Control, Cd: Cadmium, Cana: Canagliflozin, Sita: Sitagliptin, TGF−β: Transforming growth factor−β, α−SMA: Alpha-smooth muscle actin.

As shown in Figure 3, Notch1, TGF-β, SMAD3, and α-SMA relative expression in heart tissue rose considerably concomitant with a significant decline in that of SMAD7 (p < 0.001), when compared to CTRL values. However, in contrast to Cd grp, Notch1, TGF-β, SMAD3 and α-SMA, relative expression markedly decreased in both Cd + Cana (p = 0.001, <0.001, <0.001and <0.001, respectively) and Cd + Sita (p < 0.001) groups attaining the standard values except for TGF-β and α-SMA of the Cd + Sita grp (p = 0.013 and <0.001, respectively). The Cana effect on cardiac α-SMA was more favorable than that of Sita (p = 0.021). Regarding SMAD7, both therapeutics did not achieve any increment in its expression when compared to CTRL values (p < 0.001).

Likely, there was a considerable rise in hepatic tissue relative expression of Notch1, TGF-β, SMAD3 and α-SMA concomitant with a notable decrease in SMAD7 (p < 0.001) in Cd grp, when opposed to CTRL values. However, in contrast to Cd grp, Notch1, TGF-β, SMAD3 and α-SMA expression substantially decreased in both Cd + Cana (p < 0.001) and Cd + Sita (p < 0.001) groups attaining the standard values except for the α-SMA of the Cd + Cana grp (p = 0.008) and TGF-β of Cd + Sita grp (p = 0.017). While Cana effect was more favorable than that of Sita, however, no statistical difference was detected (p > 0.05). Regarding SMAD7, when contrasted to Cd grp, both therapeutics achieved a comparable increment in its expression (p < 0.001) without complete recovery when compared to CTRL values (Cd + Cana grp; p = 0.004 and Sita grp; p = 0.003).

### 3.5 Histopathological results

#### 3.5.1 H&E stain results of cardiac and hepatic sections examination

**FIGURE 4:**
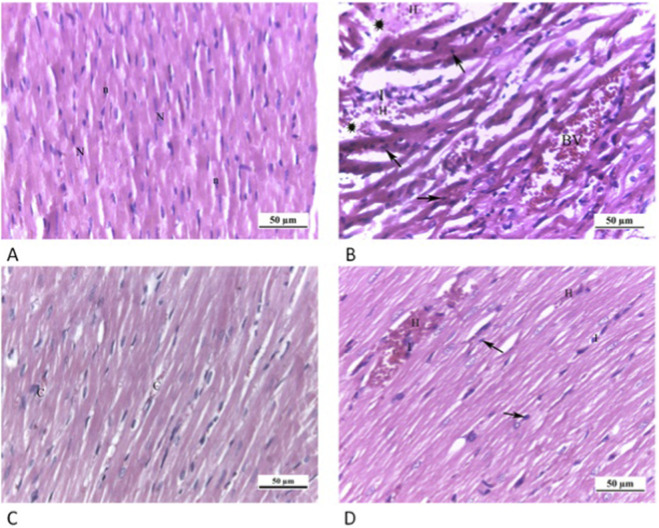
Photomicrographs of myocardium longitudinal sections: **(A)** CTRL grp shows striated branching cardiac muscle fibers with acidophilic cytoplasm and central oval vesicular nuclei (N). Connective tissue cell nuclei (n) are observed between the cardiac myocytes. **(B)** Cd grp exhibits disrupted and irregularly arranged muscle fibers. Pyknotic nuclei (arrows) along with deeply stained cytoplasm are noticed. Dilated congested blood vessels (BV), interstitial edema (asterisks), hemorrhage (H) and inflammatory cellular infiltration (I) are also observed. **(C)** Cd + Cana grp shows more or less typical heart muscle fibers pattern alongside slight blood capillary congestion **(C)**. **(D)** Cd + Sita grp shows a cardiac muscle fibers regular arrangement with some pyknotic nuclei (arrows). Hemorrhage (H) and inflammatory cellular infiltration (I) are observed among cardiac muscle fibers. (H& E ×400).

Longitudinal sections in the myocardium of all groups were examined as shown in Figure 4. Sections in rat cardiac muscle of the CTRL grp exhibited regular morphology of striated branching and anastomosing cardiac muscle fibers in various planes. Muscle fibers featured a single oval vesicular nucleus in the center, together with acidophilic sarcoplasm. Inside the narrow endomysium between myocytes, connective tissue cell nuclei were visible. However, Cd grp sections exhibited disrupted and irregularly arranged cardiac muscle fibers. Pyknotic nuclei with deeply stained cytoplasm appeared. Dilated congested blood vessels, interstitial edema, hemorrhage and infiltrate of inflammatory cells were also noticed.

Contrarily, Cd + Cana grp exhibited more or less standard cardiac muscle fibers microstructure despite slight capillaries congestion. Alike, Cd + Sita grp showed a preserved regular cardiac muscle fibers arrangement. Nevertheless, some nuclei were pyknotic. Nonetheless, there was evidence of hemorrhage and inflammatory cell infiltrates among the muscle fibers.

**FIGURE 5:**
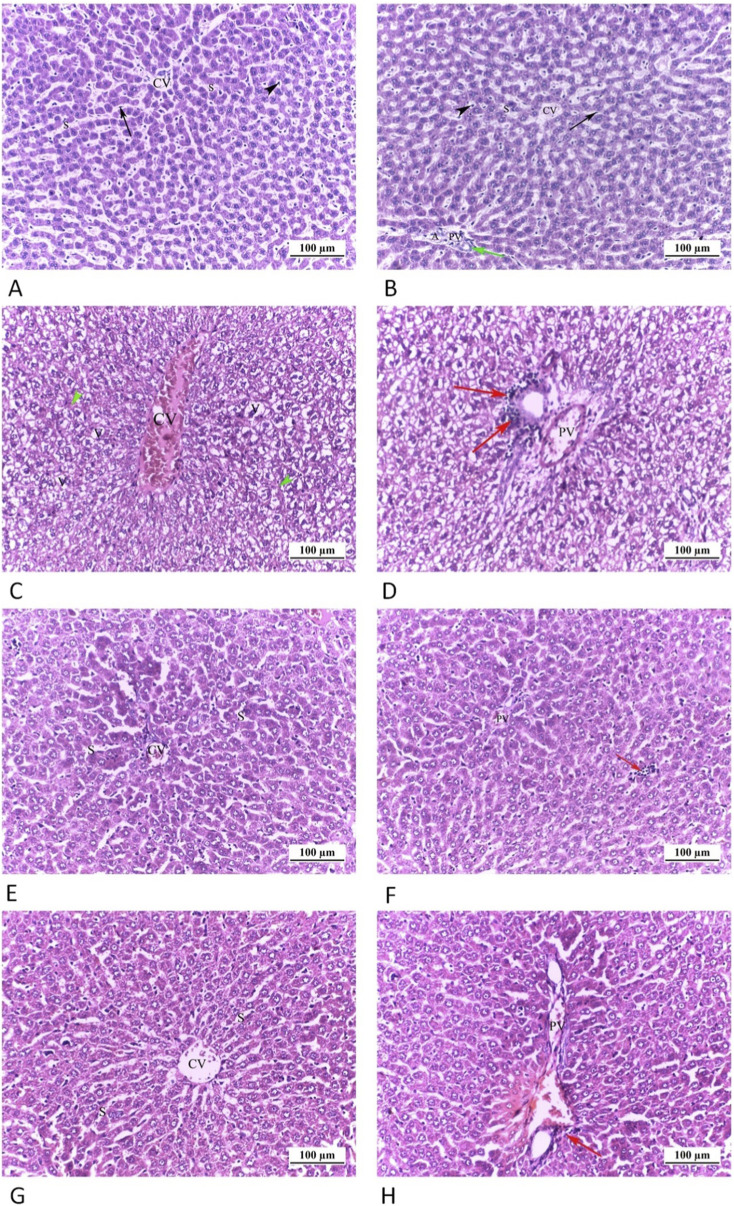
Photomicrograph of liver tissue sections: **(A,B)** CTRL grp, displaying normal hepatic cords orientated radially all around the central veins (CV) with blood sinusoids (S), lined by Endothelial (arrows) and Kupffer (arrow heads) cells, between them. The portal triad is seen including hepatic artery (H), portal vein (PV) and bile ductile (green arrow). **(C,D)** Cd grp, shows a dilated congested central vein (CV). Distortion of hepatic architecture with obliteration of sinusoids (green head arrows) is noticed. Swelling and ballooning of hepatocytes with vacuolated cytoplasm (V) are seen. D exhibits a dilated congested portal vein (PV) and periportal cellular infiltrates (red arrows). **(E,F)** Cd + Cana grp, E, shows a nearly normal hepatic architecture with preserved blood sinusoids. Slight congested central vein (CV) is seen. F exhibits slight congested portal vein (PV) and few cellular infiltrates (red arrow). **(G,H)** Cd + Sita grp, G, Hepatic cords are arranged almost normally, with blood sinusoids (S) between them. Mild dilated congested central vein (CV) is visible. H shows mild dilated congested portal vein (PV) as well as minimal periportal cellular invasion (red arrow). (H& E ×200).

On examining the H&E-stained liver sections of CTRL grp, it illustrated the usual morphological structure of the liver including typical radially directed hepatic cords circling central veins, alongside blood sinusoids (lined with endothelial and Kupffer cells) (Figure 5). Moreover, the portal triad was seen including hepatic artery, portal vein and bile ductule. Cd grp exhibited a dilated congested central vein and distorted hepatic architecture with obliterated sinusoids. Also, swelling and ballooning of hepatocytes with vacuolated cytoplasm was seen. Moreover, there were periportal cellular infiltrates and a dilated, congested portal vein. However, hepatic sections of Cd + Cana grp showed a nearly normal hepatic architecture with preserved blood sinusoids, and slightly congested central and portal veins and few cellular infiltrates were observed. Similarly, Cd + Sita grp sections revealed that the hepatic cords were arranged almost normally, with blood sinusoids between them. Nevertheless, there were little periportal cells infiltration and mildly dilated, congested portal and central veins.

#### 3.5.2 MT stain results of cardiac and hepatic sections examination

**FIGURE 6:**
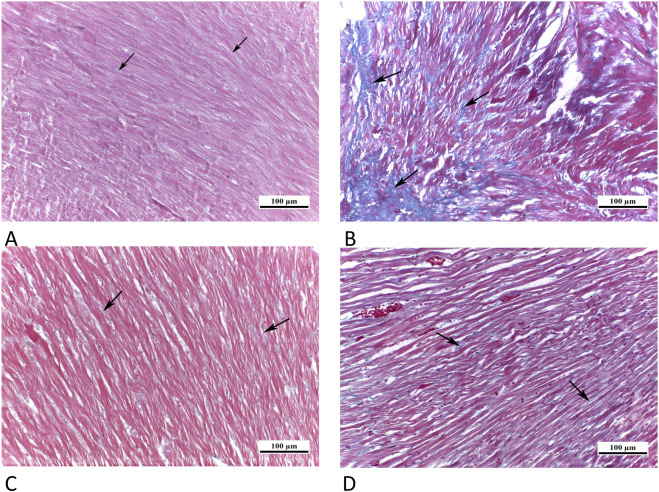
Photomicrographs of myocardial sections **(A)** CTRL grp showing tiny blue stained collagen fibers (arrows) in endomysium amongst red stained cardiac muscle fibers. **(B)** Cd grp showing greater density of collagen fibers (arrows) among cardiac muscle fibers. **(C)** Cd + Cana grp showing a minimal deposition of collagen (arrows) among cardiac muscle fibers. **(D)** Cd + Sita grp exhibiting reduced collagen amount (arrows) amid cardiac muscle fibers. (Masson’s Trichrome ×200).

Tiny, blue-stained collagen fibers were visible in the endomysium within the red-stained heart muscle fibers in sections of CTRL grp. Nonetheless, among the heart muscle fibers, Cd grp showed a greater quantity of collagen fibers. Conversely, Cd + Cana grp showed that there was only a minimal collagen fibers accumulation among the heart muscle fibers. As well, there were fewer collagen fibers among the heart muscle fibers in the Cd + Sita grp (Figure 6).

**FIGURE 7:**
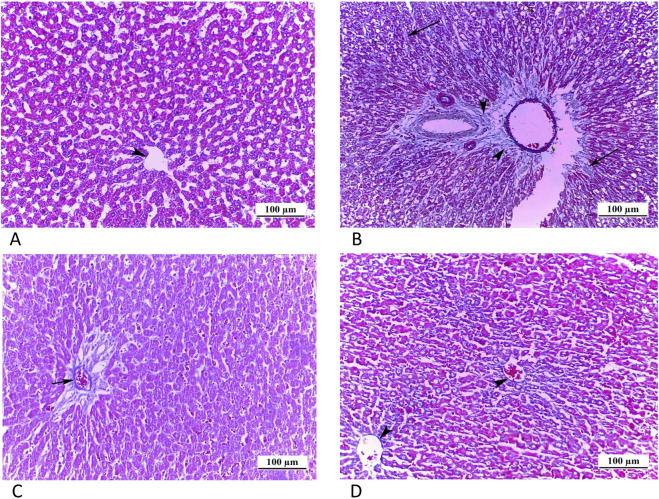
Photomicrograph of liver sections: **(A)** CTRL grp showing scanty collagen fibers (arrowhead). **(B)** Cd grp showing a substantial increment in collagen fibers in portal area (arrow heads) and between hepatic cords (arrows). **(C)** Cd + Cana grp showing few amounts of collagen deposition (arrow) in the portal area. **(D)** Cd + Sita grp showing some deposited collagen fibers (arrowheads) encompassing central vein. (Masson’s Trichrome ×200).

Liver section of CTRL grp showed scanty collagen fibers. However, Cd grp revealed a rise in collagen fibers in the portal area and among hepatic cords. Contrarily, Cd + Cana grp exhibited few amounts of collagen deposition in the portal area while Cd + Sita grp showed some deposited collagen fibers encircling the central vein (Figure 7).

#### 3.5.3 Cardiac and hepatic caspase 3 IHC results

**FIGURE 8:**
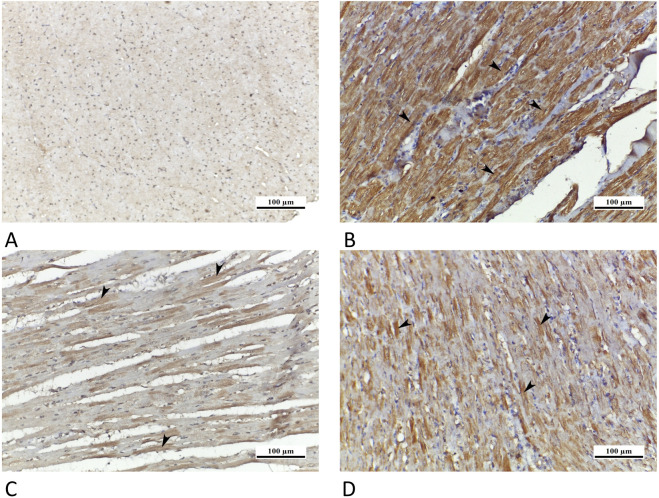
Photomicrographs of myocardial sections: **(A)** CTRL grp showing caspase 3 negative immunoreaction. **(B)** Cd grp showing caspase 3 highly positive immunoreaction (arrowheads). **(C)** Cd + Cana grp displaying caspase 3 mildly positive immunoreaction (arrowheads). **(D)** Cd + Sita grp revealing caspase 3 moderately positive immunoreaction in cardiomyocytes (arrowheads). (Caspase 3 × 200).

Sections of the cardiac muscle from CTRL grp showed a negative immunoreaction for caspase 3. A high positive sarcoplasmic immunoreaction was detected in many cardiac muscle fibers in Cd grp. Whereas Cd + Cana grp showed a mild positive sarcoplasmic immunoreaction, and Cd + Sita grp displayed a moderate positive sarcoplasmic immunoreaction (Figure 8).

**FIGURE 9:**
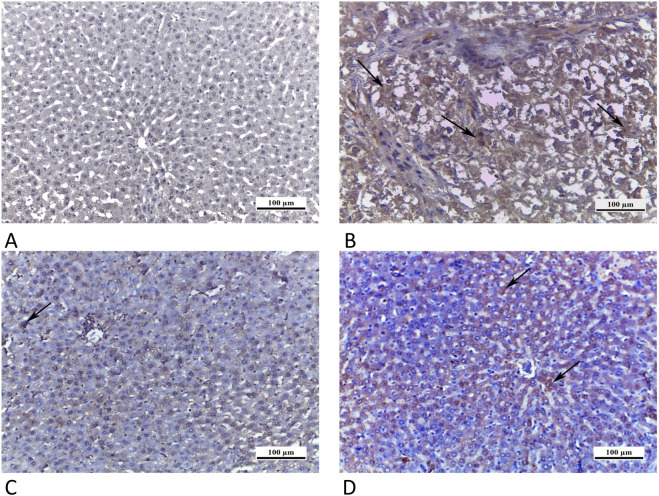
Photomicrographs of liver sections: **(A)** CTRL grp presenting a caspase 3 negative immunoreaction. **(B)** Cd grp showing enhanced caspase 3 cytoplasmic immunoreaction (arrows). **(C)** Cd + Cana grp showing mild positive cytoplasmic immunoreaction for caspase 3 (arrow). **(D)** Cd + Sita grp demonstrating caspase 3 moderately positive cytoplasmic immunoreaction (arrows). (Caspase 3 × 200).

Similarly, sections of rat liver from CTRL grp showed a caspase 3 negative immunoreaction. Cd grp revealed caspase 3 intense positive cytoplasmic immunoreaction. Whereas Cd + Cana grp showed a mild positive cytoplasmic immunoreaction, and Cd + Sita grp displayed a moderate positive cytoplasmic immunoreaction (Figure 9).

#### 3.5.4 Cardiac and hepatic NF-κB IHC results

**FIGURE 10:**
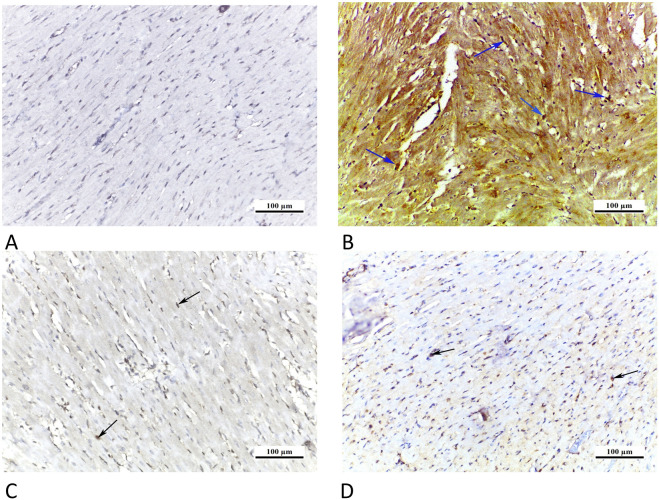
Photomicrographs of myocardial sections from different groups: **(A)** CTRL grp presenting a negative NF-κB immunoreaction. **(B)** Cd grp revealing high positive NF-κB nuclear/paranuclear immunoreaction (arrows). **(C)** Cd + Cana grp showing mild positive nuclear/paranuclear immunoreaction for NF-κB (arrow). **(D)** Cd + Sita grp exhibiting moderately positive NF-κB nuclear/paranuclear immunoreaction (arrows). (NF-κB ×200).

The CTRL grp showed a negative NF-κB immunoreaction. In Cd grp, a high positive nuclear/perinuclear immunoreaction for NF-κB was observed. On contrary, Cd + Cana grp exposed a slightly positive NF-κB nuclear/perinuclear immunoreaction. However, in Cd + Sita grp there was a moderately positive NF-κB nuclear/perinuclear immunoreaction (Figure 10).

**FIGURE 11:**
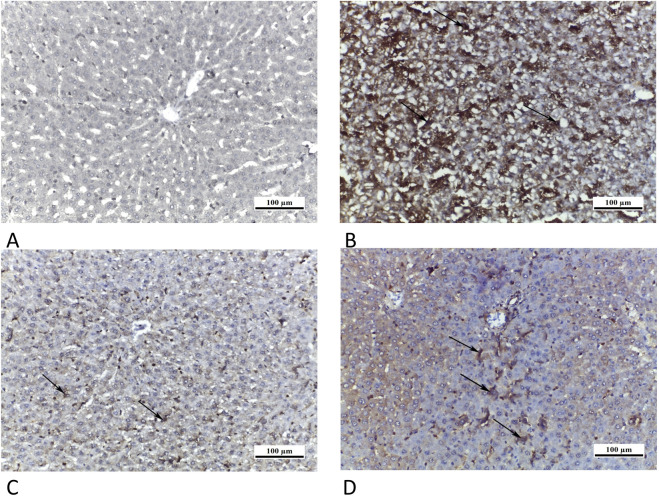
Photomicrographs of liver sections: **(A)** CTRL grp expressing a negative NF-κB immunoreaction. **(B)** Cd grp demonstrating a high positive NF-κB nuclear/paranuclear immunoreaction (arrows). **(C)** Cd + Cana grp showing mild positive nuclear/paranuclear immunoreaction for NF-κB (arrows). **(D)** Cd + Sita grp showing moderately positive nuclear/paranuclear NF-κB immunoreaction (arrows). (NF-κB ×200).

The CTRL grp showed a negative NF-κB immunoreaction while in Cd grp, a high positive nuclear/perinuclear immunoreaction for NF-κB was observed. On contrary, Cd + Cana grp displayed a mildly positive NF-κB nuclear/perinuclear immunoreaction. However, Cd + Sita grp indicated NF-κB nuclear/perinuclear immunoreactivity that was moderately positive (Figure 11).

#### 3.5.5 Cardiac and hepatic iNOS IHC results

**FIGURE 12:**
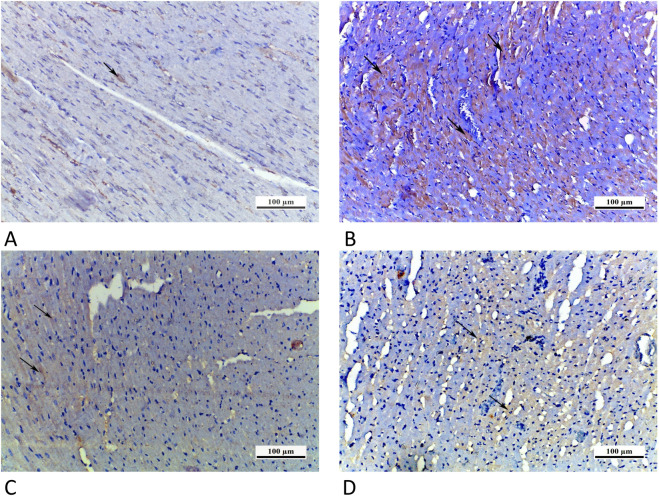
Photomicrographs of myocardial sections where cytoplasmic iNOS immunoreaction was weak (arrow) in CTRL grp **(A)**, highly positive (arrows) in Cd grp **(B)**, weakly positive (arrows) in Cd + Cana grp **(C)** and mildly positive (arrows) in Cd + Sita grp **(D)**. (iNOS ×200).

Myocardial sections of CTRL grp showed a weak sarcoplasmic immunoreaction for iNOS. Cd grp exhibited a high positive sarcoplasmic immunoreaction for iNOS in cardiac myocytes. However, a weak positive sarcoplasmic immunoreaction was observed in Cd + Cana grp. Nevertheless, Cd + Sita grp showed a mild positive sarcoplasmic immunoreaction for iNOS (Figure 12).

**FIGURE 13:**
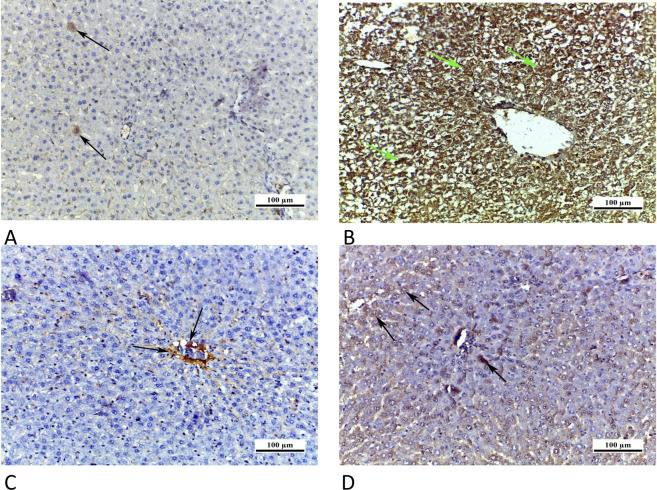
Photomicrographs of liver sections in which cytoplasmic iNOS immunoreaction (arrows) was weak in CTRL grp **(A)**, strongly positive in Cd grp **(B)**, mildly positive in Cd + Cana grp **(C)** and moderately positive in Cd + Sita grp **(D)**. (iNOS ×200).

Liver sections of CTRL grp revealed a weak cytoplasmic iNOS immunoreaction. Cd grp exhibited a high positive cytoplasmic immunoreaction for iNOS. However, a slightly positive cytoplasmic iNOS immunoreaction was visible in Cd + Cana grp versus a modest positive cytoplasmic iNOS immunoreaction in Cd + Sita grp (Figure 13).

#### 3.5.6 Cardiac and hepatic histomorphometry results

**FIGURE 14:**
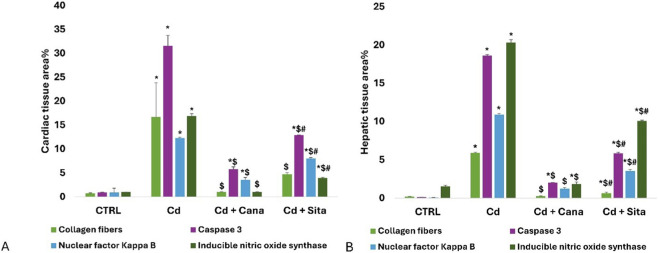
Bar charts present quantitative analysis of cardiac **(A)** and hepatic **(B)** tissues’ collagen fibers, caspase 3, NF-κB and iNOS area % among different groups. Data was depicted as means± standard deviations. (n = 6). Statistical significance was determined by one-way ANOVA followed by Tukey’s *post hoc* test. *p* ≤ 0.05 is significant. *: Significant vs. CTRL, $: Significant vs. Cd, #: Significant vs. Cd+Cana. CTRL: Control, Cd: Cadmium, Cana: Canagliflozin, Sita: Sitagliptin.

As shown in Figure 14A, the cardiac tissues’ mean area percentage (%) of collagen fibers, caspase 3, NF-κB, and iNOS in the Cd grp increased significantly (p < 0.001) compared to CTRL. Whereas relative to Cd grp, these percentages significantly improved (p < 0.001) in Cd + Cana and Cd + Sita groups without attaining CTRL percentages except for (collagen and iNOS) % that were almost normalized in Cd + Cana versus complete recovery of collagen% only in Cd + Sita groups (p > 0.05). Worth mentioning, Cana’s impact was noticeably better than Sita’s (p < 0.001) on all areas %, except for collagen their favorable effect was almost comparable (p > 0.05).

As revealed in Figure 14B, liver tissues’ mean area % of collagen, caspase 3, NF-κB, and iNOS in Cd grp increased significantly (p < 0.001) compared to CTRL. Whereas relative to Cd grp, these percentages significantly reduced (p < 0.001) in Cd + Cana and Cd + Sita groups without attaining CTRL percentages except for the collagen % and iNOS % that were almost normalized in the Cd + Cana grp (p > 0.05) reflecting the potency of Cana as antifibrotic and anti-nitrosative stress therapeutic on the liver as well as the heart as mentioned earlier. On all area percentages, Cana’s outcome was substantially greater than Sita’s (p < 0.001).

## 4 Discussion

Our research demonstrated that long-term consumption of Cd caused significant hepatic and cardiac damage, as proven by elevated blood AST, ALT, LDH, CK-MB, cTnI and cTnC with decreased plasma albumin levels. Also, cardiac and hepatic inflammation was confirmed by the rise in IL-1β, TNF-α concomitant with decline in IL-10 level in addition to increased immunoreactivity of cardiac and hepatic tissues NF-kB. Moreover, the hepatic and cardiac tissues of the Cd grp rats showed elevated OS activity (decreased GSH with rise in MDA) and nitrative stress (rise in iNOS immunoreactivity), fibrotic changes detected with MT staining and confirmed by the increase in Notch1, TGF-β, SMAD3, and α-SMA relative expression in parallel with decrease in SMAD7 relative expression in both tissues. Also, elevated caspase 3 immunoreactivity verified the cardiac and hepatic apoptotic changes. Nevertheless, both Cana and Sita displayed comparable (p > 0.05) improvement in the serum ALT/AST, cardiac/hepatic MDA and GSH, and mRNA expression levels of Notch1, TGF-β, and SMAD3/7. However, Cana exhibited a superior protective effect (p < 0.05) than Sita regarding the hepatic collagen%, cardiac and hepatic % of caspase 3, NF-κB and iNOS. Importantly, statistical analysis revealed no significant fluctuations in blood glucose levels among all the experimental groups, confirming the normoglycemic nature of the model.

### 4.1 Cardio-hepatic toxic effects following chronic Cd exposure

There is conflicting evidence regarding Cd-induced hyperglycemia. The effects may vary depending on the species and period of exposure to Cd. Literature indicates that Cd exposure can cause hyperglycemia or impaired glucose tolerance by destroying pancreatic beta cells (El Muayed et al., 2012; Chang K.-C. et al., 2013; Wong et al., 2021). On the contrary, our findings-where blood glucose levels did not significantly change-align with research studies centered on the early-to-mid stages of chronic Cd toxicity in normoglycemic models (Barregard et al., 2013; Li X. et al., 2019; Satarug, 2023) with the claim that Cd exposure does not reduce the tissue sensitivity to insulin (Li X. et al., 2019). However, this requires thorough investigation. Worth noting, the maintained glucose levels in our study supports the presence of other mechanisms driving the cardio-hepatic damage upon Cd exposure rather than metabolic disorders including diabetes.

Consistent with our results, Ibrahim and Ahmed discovered that following Cd intoxication, there was a marked rise of LDH, CM-KB and AST in serum, which are markers of myocardial injury (Ibrahim Fouad and Ahmed, 2022), and impaired liver functions (Fan et al., 2018). Additionally, Cd exposure caused poorly organized sarcomeres and myofibrils, perivascular and interstitial fibrosis, and cardiomyocyte apoptosis with elevated expression rates of matrix metalloproteinase-2/14 in cardiac tissues (Ghosh and Indra, 2018; Shen et al., 2018; Chou et al., 2023).

Cd exhibits a strong propensity for GSH, which is crucial for defense mechanisms towards ROS and detoxification of xenobiotic substances (Rao et al., 2025), and disrupts its normal function (Genchi et al., 2020). Additionally, Cd reduces the cardiac expression of master antioxidants; nuclear factor erythroid 2-related factor 2 (Nrf2)/heme oxygenase 1 genes (Refaie et al., 2019; Habib et al., 2022). Consequently, Cd causes lipid peroxidation via provoking either ROS avalanche (Sarkar et al., 1995; Das and Al-Naemi, 2019) and/or iNOS triggered reactive nitrogen species generation (Cannino et al., 2009; Cuypers et al., 2010) in the heart and liver (Yazihan et al., 2011; Alruhaimi et al., 2023).

Furthermore, the OS caused by Cd causes cell apoptosis by directly boosting mitochondrial membrane’s permeability, which permits release of cytochrome C. This activates caspase 8, which subsequently triggers B-cell lymphoma 2 (Bcl-2) associated X-protein (Bax) and cleaved caspase 3 over Bcl-2 in the cardiac (Ghosh and N, 2018) and hepatic (Thévenod and Lee, 2013; Amamou et al., 2015) tissues.

Additionally, cyclo-oxygenase 2 (COX2), iNOS, and Toll-like receptors-4 (TLR-4)/NF-κB are activated by Cd, thereby contributing to increased proinflammatory cytokines, specifically TNF-α, IL-6, and IL-1β, which ultimately cause tissue inflammation (Chen et al., 2021; Wang Z. et al., 2021) provoking more ROS production, thus creating an endless oxido-inflammatory circuit in the cardiac (Fan et al., 2021) and hepatic (Liu et al., 2024) tissues. Furthermore, excessive iNOS, TNF-α, and IL-1 expression stimulates p53, NF-κB, and hypoxia inducible factor (HIF)-1α, which subsequently trigger more inflammatory chemokines/cytokines formation (Reuter et al., 2010).

The occurrence and progression of cardiac interstitial fibrosis upon Cd exposure, are mostly attributed to TGF-β which promotes myofibroblast differentiation and ECM deposition, evident with collagen and α-SMA overexpression and accumulation. Furthermore, TGF-β1 can cause inflammation and exacerbate the generation of ROS (Kong et al., 2023; Li, 2025) which are drivers for profibrogenic TGF-β1 expression, collagen accumulation, and hepatic stellate cells (HSCs) activation (Garbuzenko, 2022). Based on our knowledge, the modulation of TGF-β/SMAD pathway in the heart following Cd exposure has not yet been investigated. However, in agreement with our findings, TGF-β/SMAD pathway was documented to mediate Cd-triggered liver fibrosis and EMT in chicken (Zhang et al., 2025).

Notch mediates many human fibrotic diseases (Morell et al., 2013; Zheng et al., 2013). Liver mesenchymal/epithelial compartments extensively express Notch proteins. In α-SMA-positive myofibroblasts of damaged livers, Jagged 1, a Notch ligand, is markedly upregulated, which causes hepatic Notch activation and copious collagen matrix synthesis (Morell et al., 2013) and liver fibrosis (Chen et al., 2012; Hu and Phan, 2016). The fibrogenic attributes of TGF-β1 are preferentially mediated by Notch signalling, which in turn promotes the transformation of HSCs into myofibroblasts upon liver damage as well as the production and accumulation of ECM elements like collagen I, α-SMA, and glial fibrillary acidic protein (Morell et al., 2013; Zheng et al., 2013; Tanriverdi et al., 2016). In alignment with our results, Cd induced fibrogenic changes in hepatocellular carcinoma cells by activating Notch pathway (Zhang et al., 2025).

Similarly, ROS avalanche driven on by Cd fosters the induction of P38 mitogen-activated protein kinase (MAPK) and phosphoinositide 3-kinase (PI3K)- protein kinase B (PKB) axes, which results in heart injury and fibrosis (González et al., 2018; Shen et al., 2018). Moreover, in cardiac fibrosis, TGF-β and Notch paths operate mutually and synergistically (Russell et al., 2011). Nevertheless, the modulation of Notch and its implication in cardiac fibrosis was not investigated before in the context of chronic Cd exposure. Our study is the foremost research which revealed that cardiac fibrosis is associated with Notch1 modulation in chronic Cd exposure model. Therefore, based on our findings, Notch 1 inhibitors could efficiently manage the cardiac and hepatic fibrosis spurred on by Cd.

Collectively, discovering novel prophylaxis with an amalgam of antioxidant, anti-inflammatory, antiapoptotic, and antifibrotic properties to intercept or mitigate chronic Cd-induced cardiac and hepatic damage is crucial since exposure to chronic Cd toxicity is ubiquitous.

### 4.2 Protective cardio-hepatic effects of Cana independent of glycemic modulation

It has been established that cardiomyocytes and cardiac endothelial cells express SGLT2, and that this expression rises with heart failure (Marfella et al., 2022; Mroueh et al., 2023). In support of our findings, dapagliflozin considerably lessened infarct size and reduced serum LDH, CK MB, and cTnI in myocardial ischemia/reperfusion model, irrespective of its glycemic impact (Zhang et al., 2025). Although the liver expresses SGLT2, chronic liver illness does not cause a rise in its expression (Nakano et al., 2022). Nevertheless, SGLT2is led to reduction in liver enzyme levels and improvement in liver functions and structure (Bellanti et al., 2022) indicating that the Cana dose was effective prophylaxis without being hepatotoxic as confirmed in the current study.

Corroborated with our findings, Cana exhibited minor non-significant impacts on blood glucose levels in normoglycemic circumstance proving that it is well-tolerated and did not induce hypoglycemia in non-diabetic rats. Literature reported that Cana decreases renal glucose reabsorption by 55% in normoglycemic rats without inducing hypoglycemia, while its inhibitory effect increases to approximately 94% in hyperglycemic rats (Kuriyama et al., 2014). This could be attributed to the inability of SGLT2i to increase insulin secretion. Further, despite SGLT2 inhibition, compensatory mechanisms such as sodium glucose transporter 1 reabsorption and hepatic glucose synthesis contribute to the maintenance of physiological plasma glucose levels (Fonseca-Correa and Correa-Rotter, 2021). Thus, other molecular mechanisms-beyond glycemic control-definitely impact the SGLT2is′ hepatic and cardiovascular implications (Hu et al., 2024). Among the underlying mechanisms, SGLT2is reduce the production of ROS by acting as indirect antioxidants. For example, empagliflozin blocks the Na+/H+ exchanger (NHE), PKB/mammalian target of rapamycin (mTOR), and sirtuin1/AMP-activated protein kinase signalling (AMPK) thereby lowering ROS (Mizuno et al., 2018), therefore improving the cardiac performance (Zhou H. et al., 2018; Koizumi et al., 2023). In conformity with our findings, dapagliflozin decreased lipid peroxidation and enhanced GSH in the cardiac and hepatic tissues by upregulating sirtuin 6 protein expression (Ma et al., 2024) and PI3K/PKB/mTOR (Zhang et al., 2025), respectively. Furthermore, Cana enhances the antioxidants’ activity of superoxide dismutase (SOD) and GSH peroxidase (GPx) (Kabil and Mahmoud, 2018) by activating AMPK, PKB, and endothelial nitric oxide synthase while inhibiting iNOS and NADPH oxidase 4 (Hasan et al., 2020b). Though these outcomes strongly endorse the antioxidant significance of SGLT2is, precise measures of cardiac and hepatic GSH and MDA in chronic Cd-induced toxicity have not yet been investigated.

Similar to our findings, SGLT2is have been documented to reduce cardiac inflammation, a key culprit of heart fibrosis (Smolgovsky et al., 2021), by reversing HIF-2α signalling, blocking phosphorylation of IκB kinase, and suppressing NF-κB p65 transcription (Yang et al., 2022; Lv et al., 2024), and MyD88-related pathways (Quagliariello et al., 2021), activating AMPK with downstream inhibition of the NHE-1 therefore, reducing: nucleotide-binding domain, leucine-rich–containing family, pyrin domain–containing-3 inflammasome and p38-dependent toll-like receptor 4 expression (Hu et al., 2023), IL-1β and TNF-α, besides ameliorating mitochondrial ROS release (Lee et al., 2022). Additionally, SGLT2is increase macrophages (M2 phenotype) differentiation and decrease iNOS expression in a mechanism that is reliant on AMPK (Koyani et al., 2020). Alike, empagliflozin improved liver inflammatory and fibrotic changes in non-alcoholic steatohepatitis rats by hitting hepatic NF-κB (Elseweidy et al., 2024).

Further, Cana therapy decreased Bax/Bcl-2 ratio and cleaved caspase 3- a typical indicator of apoptosis-in cardiac cells (Long et al., 2022). Likely, Cana can increase the expression of hepatic Bcl-2 and decrease serum caspase 3 levels thereby halting hepatocyte apoptosis (Thapaliya et al., 2014; Kabil and Mahmoud, 2018). Throughout fibrosis, endothelial cells become more dynamic and express fibroblast-specific protein 1 (FSP1) and α-SMA. This promotes the growth of fibroblasts and myofibroblasts in the cardiac tissue and results in an overabundance of ECM elements including collagen in a process termed “Endothelial-to-mesenchymal transition (EndMT)” (Choi et al., 2020). SGLT2is were discovered to prevent myocardial fibrosis by hampering EndMT through: AMPKα-dependent downregulation of TGF-β/SMAD (Tian et al., 2021), sirtuin 1 stimulation (Wang et al., 2023), and TGF-β-activated kinase 1, NF-κB phosphorylation (Zhang et al., 2023), and NHE-1 (Rolski and Mączewski, 2025) inhibition as well as the direct interaction with sodium–myoinositol and sodium–multivitamin cotransporters (Chung et al., 2023; Schmidt et al., 2024). By inhibiting mTOR, SGLT2is can also promote regulatory T cells differentiation while decreasing T helper (Th)1/17 differentiation, which is another anti-fibrotic strategy (Qin et al., 2022). Additionally, SGLT2is shift the metabolism from glucose to lipids, boosting ketones generation, which are effective heart fuel, particularly under stress, and may lessen OS and fibrosis (Saucedo-Orozco et al., 2022).

Likewise, clinical data demonstrated that SGLT2is help to normalize liver functions and lessen fibrosis owing to decreased hepatic myeloperoxidase formation as well as oxidative protein carbonyl groups and thiobarbituric acid-reactive compounds (Tahara et al., 2014; Nakano et al., 2015), ROS (Tang et al., 2017), and TNF-α, IL-6 and monocyte chemoattractant protein-1 expression (Youssef et al., 2023).

In alignment with our data, Shen et al. found that empagliflozin reduces hepatic fibrosis by blocking the TGF-β pathway in HSCs secondary to miR-34a-5p downregulation (Shen et al., 2023). Furthermore, our findings demonstrated that Cana might upregulate SMAD7, an adverse TGF-β signalling pathway regulator, while downregulating Notch1/TGF-β/SMAD3 which contributes to cardiac (Bakalenko et al., 2024) and hepatic (Dooley et al., 2003; Wang et al., 2017) fibrosis. However, no current literature supports these results and comprehensive research in this scope is required.

### 4.3 Protective cardio-hepatic effects of Sita independent of glycemic modulation

Dipeptidyl peptidase 4 (DPP4) inhibitors (DPP-4is), like Sita, improve the glycemic index by increasing the release of incretin and glucagon-like peptide-1 (GLP-1) thus enhancing insulin production and suppressing glucagon “solely when glucose levels are elevated” (Scheen, 2012) without resulting in hypoglycemia (Karagiannis et al., 2014; Ahrén, 2019) as reported in our results. The ability of Sita to decrease the AST, ALT, LDH, CK-MB, cTnI and cTnC and increase albumin levels in blood, reflects its cardio-hepatic protective effects as emphasized in previous studies (Chang G. et al., 2013; Al-Rasheed et al., 2016; Zhou Y. et al., 2018; Maleki et al., 2024). This highlights the necessity for investigating the additional underpinning molecular pathways by which DPP-4is, particularly Sita, can attenuate cardiac or hepatic impairments beyond their hypoglycemic effect.

The heart and immune cells abundantly express the DPP4 (Zhong et al., 2015). The DPP4 activity is frequently linked to cardiac remodeling and inflammation (Esposito et al., 2017). Through T-cell co-stimulation, macrophage maturation, and inflammatory cell adherence to ECM proteins, DPP4 enhances the inflammatory process (Zhong and Rajagopalan, 2015).

Our study revealed that Sita lowered the cardiac MDA while increased the GSH and reduced the NF-κB, iNOS and caspase 3 expression resulting in refined oxido-inflammatory status in support to prior results (Al-Rasheed et al., 2016; Sun et al., 2021; Zakaria et al., 2022; Alqahtani et al., 2025). Potentiation of GLP-1/GLP-1 receptor axis is probably responsible for at least some of Sita’s cardioprotective properties (Poornima et al., 2008). GLP-1 reduces the monocyte activity, boosts the M2-polarization, and hinders the NF-κB activation (Matsubara et al., 2012; Brenner et al., 2015). Additionally, Sita’s cardioprotective properties have been attributed to a reduction in Rho-associated coiled-coil containing protein kinase 2 and Janus kinase/signal transducer and activator of transcription pathway signalling, which link to AMPK and MAPK trajectories (Al-Rasheed et al., 2016). Moreover, 15-epi-lipoxin A4, a powerful anti-inflammatory intermediary (Zhou Y. et al., 2018) and reversal of myocardial TNF-α and IL-6 (Esposito et al., 2017) were directly promoted by Sita.

Also, Sita suppresses the ROS generation and COX2 expression via triggering the GLP-1/AMPK/uncoupling protein 2 pathway (Liu et al., 2014). DPP-4 activity, 3-nitrosyin, and iNOS were found to positively correlate in cardiac patients; this association was negated by Sita, indicating Sita’s anti-nitroxidative stress action on the heart (Zhou Y. et al., 2018). Furthermore, Sita decreases Bax/Bcl-2 and cleaved/total caspase 3 ratio, which lessens OS and apoptosis. It also increases PI3K/PKB pathway signalling and Bad phosphorylation (Chang G. et al., 2013). Likewise, Sita’s hepatoprotective effect has been documented before, apart from its ability to regulate the blood glucose level (Onoyama et al., 2015; Xu et al., 2017). OS causes IκB kinase α or β to phosphorylate IκBs, which release NF-κB and drive its translocation to the nucleus. Following its binding to DNA, NF-κB increases the transcription of several inflammatory cytokines and chemokines (Zhu and Fung, 2000). Furthermore, NF-κB activation is triggered by the substantial levels of nitric oxide (NO) produced by TNF-α-driven iNOS, which combine with superoxide anions to generate the potent reactive peroxynitrite (El-Sheikh et al., 2015) causing severe oxidative damage of hepatic tissue (Mukherjee et al., 2013). Nevertheless, Sita scavenges ROS by increasing the Nrf2 expression (Choi et al., 2015) and the activity of GSH and SOD (Jung et al., 2014), thus limiting NF-κB activation and effectually alleviating liver injury and hepatotoxicity as evidenced by decreased serum ALT, AST, LDH, TNF-α, IL-1β/6, COX2 and iNOS (Lin and Lin, 2016; Wang X. et al., 2021). As previously reported, iNOS expression and NO release were suppressed by Sita (Abo-Haded et al., 2017). Additionally, OS directs Bax toward the outer mitochondrial membrane, increasing its permeability to release cytochrome C, leading to caspases activation (Zhou et al., 2024). In contrast, Sita may inhibit the hepatic tissue’s caspase 3 activity and Bax expression and level (Abo-Haded et al., 2017).

After Sita was administered, Esposito observed a decrease in cardiac TGF-β, SMAD3, connective tissue growth factor, collagen and fibronectin, impeding the development of fibrosis (Esposito et al., 2017) which supports our findings. Further, our findings revealed modulation of the Notch1 expression associated with Sita administration. It is noteworthy that Sita has been shown to ameliorate renal fibrosis and decrease ECM buildup by repressing TGF-β1/SMAD3 and expressing SMAD7 (Wang D. et al., 2018). Despite, Sita has not been verified to regulate the Notch1 in the heart, but there is significant proof that it ameliorates cardiac fibrosis by suppressing TGF-β/SMAD2/3 signaling and boosting antifibrotic SMAD7 (Hong et al., 2017; Wójcicka et al., 2023; Esposito et al., 2017).

Likewise DPP-4 is involved in fibroblast proliferation and hepatocyte ECM interactions (Brill et al., 2002). Therefore, DPP-4is may have an antifibrotic influence on hepatic fibrosis by decreasing hepatic TGF-β1 production, phosphorylating SMAD2/3, lowering total collagen, and a1(I)-procollagen expression, attenuating α-SMA, and suppressing extracellular signal-regulated kinase 1/2 phosphorylation (Kaji et al., 2014; Sueyoshi et al., 2024). p38 MAPK controls the expression of a1(I)-collagen gene in HSCs. However, it was discovered that DPP-4i interferes with the phosphorylation of p38 MAPK in activated HSCs (Tsukada et al., 2005). In a recent study, Saxagliptin, a DPP-4i, suppressed the Notch1 signaling in liver cirrhosis and hepatocellular cancer (Abd Elhameed et al., 2021). Accordingly, DPP-4is may indirectly alter Notch1-mediated fibrogenic signaling by inhibiting TGF-β/SMAD2/3 in stellate cells. Nevertheless, direct studies relating sitagliptin, Notch1, and hepatic fibrosis are still required.

### 4.4 Comparative analysis clarifying Cana’s better outcomes relative to Sita

Collectively, our investigation found that early Cana or Sita administration led to significant cardio-hepatic benefits by modifying the Notch1/TGF-β/SMAD3/7 signaling system, caspase 3, NF-κB, and iNOS, as well as OS homeostasis, rather than through metabolic adjustments. Cana outperformed Sita on blood CK-MB and cTnC levels, cardiac α-SMA and hepatic collagen area%, as well as cardiac/hepatic caspase 3%, NF-κB%, and iNOS% in addition to restoring both cardiac and hepatic architecture. Similar to our findings, Cana provided a stronger tissue-protective effect than Sita, effectively reducing injury, fibrosis, inflammation, and oxidative/nitrative stress, as evidenced by its superior effects on various biomarkers and histology (Fu et al., 2024; Sabe et al., 2024). Literature revealed that SGLT2is can lower myocardial MDA by 60% and increase GSH recovery by 80%. It also reduces NF-κB, iNOS, and caspase-3 levels more, providing better tissue protection in the heart and liver. DPP-4is, on the other hand, have less potent antioxidant, anti-inflammatory, and antiapoptotic properties (Hussein et al., 2020; Gager et al., 2021; Elrakaybi et al., 2022; Chen et al., 2024). Furthermore, this disparity in outcomes could be owed to the distinctive pharmacodynamics of SGLT2is, which have been demonstrated to directly inhibit NHE1 in the heart and lower NLRP3 inflammasome activation more effectively than DPP-4is (Yu et al., 2022). Also, the enhanced repression of nitrative stress, as indicated by the Cana group’s decreased iNOS immunoreactivity, is most likely responsible for the superior conservation of the tissue’s architecture (Hasan et al., 2020b). Moreover, in myocardial ischemia, Cana led to better heart function and cardiorespiratory performance than Sita, indicating a distinct advantage in both, the functional and structural remodeling (Carbone et al., 2020; Sabe et al., 2024). Also, SGLT2is outperformed DPP-4is in regard to cardiorenal and fatty liver outcomes, implying a higher protective impact against fibrosis and organ derangement and dysfunction (Suzuki et al., 2024b; 2024a). This is consistent with the observed decreases in hepatic collagen area % and improved hepatic histology linked with Cana in our study.

### 4.5 Strengths and limitations of the study

The current study has some noteworthy strengths. First, using a normoglycemic Wistar rat model, we consistently demonstrated that Cana and Sita’s cardio-hepatic protective actions are diverse and glucose independent. This conveys significant evidence for the possible clinical application of these medicines in non-diabetic patients subjected to environmental toxins such as Cd. Second, a juxtaposed assessment of an SGLT2i and a DPP-4i in a toxicological environment reveals a clear difference in their respective efficacies, with Cana demonstrating greater capability to preserve tissue architecture while mitigating fibrosis. Furthermore, the utilization of a diverse set of biomarkers, ranging from systemic enzymes to molecular gene expression and quantitative histomorphometry, increases the legitimacy of our findings.

Nevertheless, some limits must be addressed. Although we detected significant alterations in the Notch1/TGF-β/SMAD3 axis, our research did not include functional tests like sequencing or pharmacological inhibitors to confirm that these pathways are the primary culprit of the spotted protection. We assessed mRNA levels and protein immunoreactivity, but we did not analyze phosphorylation (e.g., p-SMAD3 or p-NF-κB) to assess their active signaling states. Also, the functions of other putative routes, such as TLR4 or the Bax/Bcl-2 ratio, were considered in this work based on prior research rather than actual measurement, thus they remain hypothetical.

### 4.6 Recommendations and perspectives

To conclusively determine the critical role of Notch1 in Cd-mediated diverse-organ damage, we should utilize specific inhibitors or genetic silencing, complemented by protein-level quantification of activated SMAD complexes via Western blot techniques to verify transcriptional results. Building on this, prospective studies evaluating the long-term safety and effectiveness of Cana and Sita in various Cd exposure models are required while investigating the precise molecular pathways underlying their protective impacts. Subsequent trials should incorporate different dosages of Cana and Sita to figure out the minimum safe concentration necessary to achieve organ protection in non-diabetics, alongside more studies about the prospects for non-glycemic benefits and possible mediating mechanisms in different tissues and pathological conditions. Furthermore, investigating the possibilities of combination therapies that combine Cana or Sita with other compounds may improve the protective properties towards organ damage caused by Cd, providing a more thorough approach to treatment. It is also worthwhile to carry out clinical studies to assess the significance of these outcomes in human populations exposed to Cd, especially in high-risk environmental or occupational circumstances. Ultimately, public health programs should include Notch1/TGF-β axis molecular markers in occupational screening for the detection of early-stage silent tissue damage prior to irreparable fibrosis.

## 5 Conclusion

Our study emphasizes how Cana and Sita might avert cardiac and hepatic damage caused by chronic Cd exposure. Despite the fact that clinical diagnosis of Cd toxicity often occurs years later, our landmark study demonstrated that early intervention with SGLT2 or DPP-4 inhibitors showed considerable cardio-hepatic improvements via disrupting the progression from initial OS to terminal fibrosis rather than through systemic metabolic improvements. By adjusting the Notch1/TGF-β/SMAD3/7 signaling system, caspase 3, NF-κB and iNOS, both regimens successfully reduced the OS, inflammation, apoptosis, and fibrosis. However, Cana demonstrated greater improvement in serum CK-MB and cTnC, cardiac α-SMA and hepatic collagen area% as well as cardiac and hepatic % of caspase 3, NF-κB and iNOS with recovery of the cardiac and hepatic architecture. This shows that, while both medications give considerable cardio-hepatic protection against Cd toxicity, the SGLT2is might provide an extra benefit in preserving tissue architecture which necessitate further investigations. Ultimately, this provides a biological basis for exploring these drugs as “off-target” protective therapies for populations at high risk of chronic environmental or occupational toxin exposure irrespective of the fact that the main culprit for organ damage was a toxin (Cd), rather than a diabetic condition.

## Data Availability

The original contributions presented in the study are included in the article/supplementary material, further inquiries can be directed to the corresponding author.
