# Correction: Beyond glycemic control: the cardiac and hepatic benefits of SGLT2 and DPP-4 inhibitors in mitigating chronic cadmium-induced inflammation, oxidative/nitrative stress, apoptosis and fibrosis

**DOI:** 10.3389/fphys.2026.1904774

**Published:** 2026-07-07

**Authors:** Fatma E. Hassan, MennaAllah M. Hassanien, Asmaa Selmy, Lamiaa Mohamed Mahmoud, Amal Darwish, Basant A. Aldreny

**Affiliations:** 1 Medical Physiology Department, Kasr Alainy, Faculty of Medicine, Cairo University, Giza, Egypt; 2 Department of Physiology, General Medicine Practice Program, Batterjee Medical College, Jeddah, Saudi Arabia; 3 Anatomy and Embryology Department, Faculty of Medicine, Cairo University, Giza, Egypt; 4 Department of Medical Biochemistry and Molecular Biology, Faculty of Medicine, Ain Shams University, Cairo, Egypt

**Keywords:** cadmium, DPP-4i, heart, liver, SGLT2i

There was a mistake in the placement of **Figures 6**–**13** under their relevant captions as published. The corrected **Figures 6**-**13** appear below.

**Figure 6:**
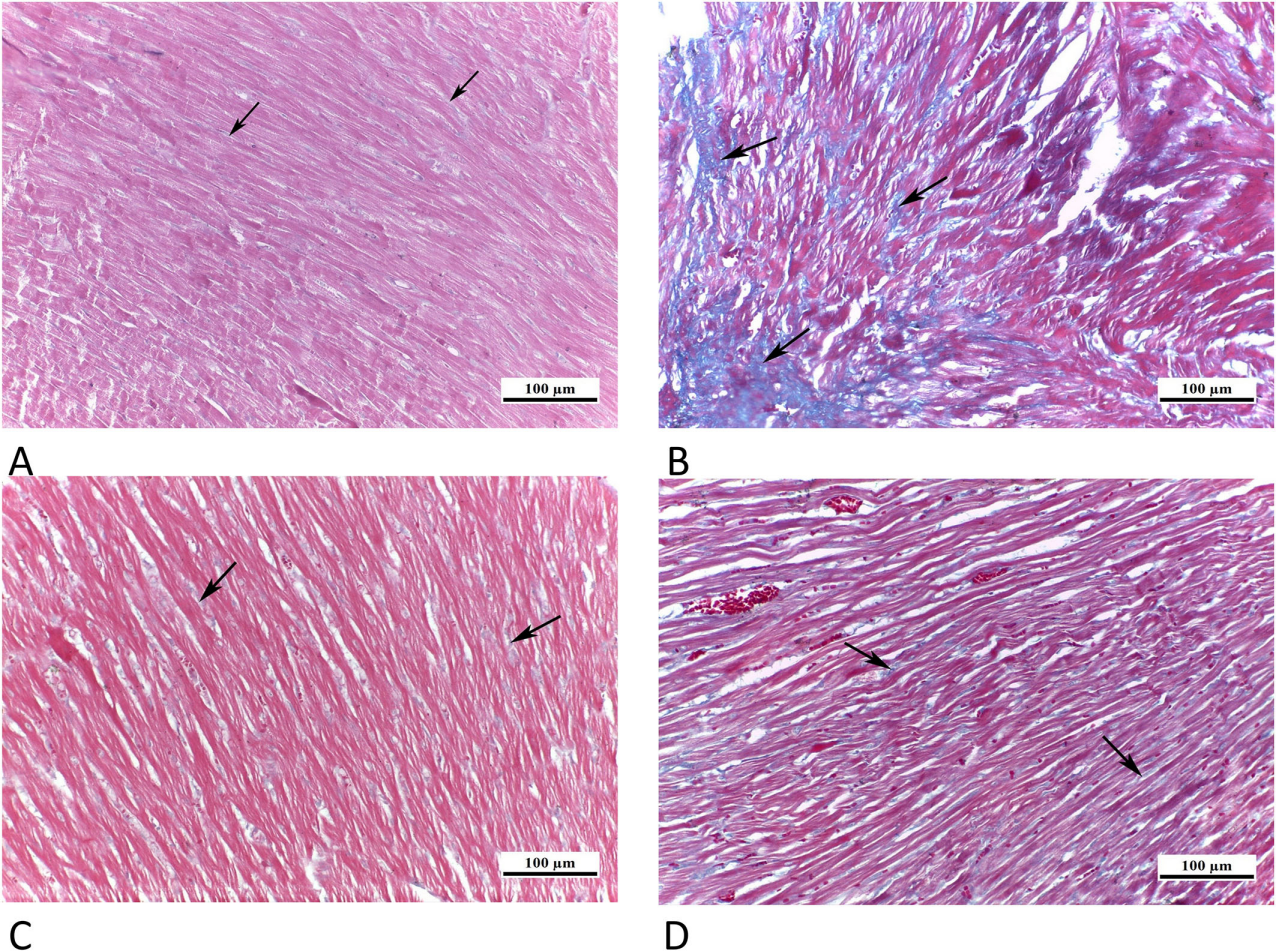
Photomicrographs of myocardial sections **(A)** CTRL grp showing tiny blue stained collagen fibers (arrows) in endomysium amongst red stained cardiac muscle fibers. **(B)** Cd grp showing greater density of collagen fibers (arrows) among cardiac muscle fibers. **(C)** Cd + Cana grp showing a minimal deposition of collagen (arrows) among cardiac muscle fibers. **(D)** Cd + Sita grp exhibiting reduced collagen amount (arrows) amid cardiac muscle fibers. (Masson’s Trichrome ×200).

**Figure 7:**
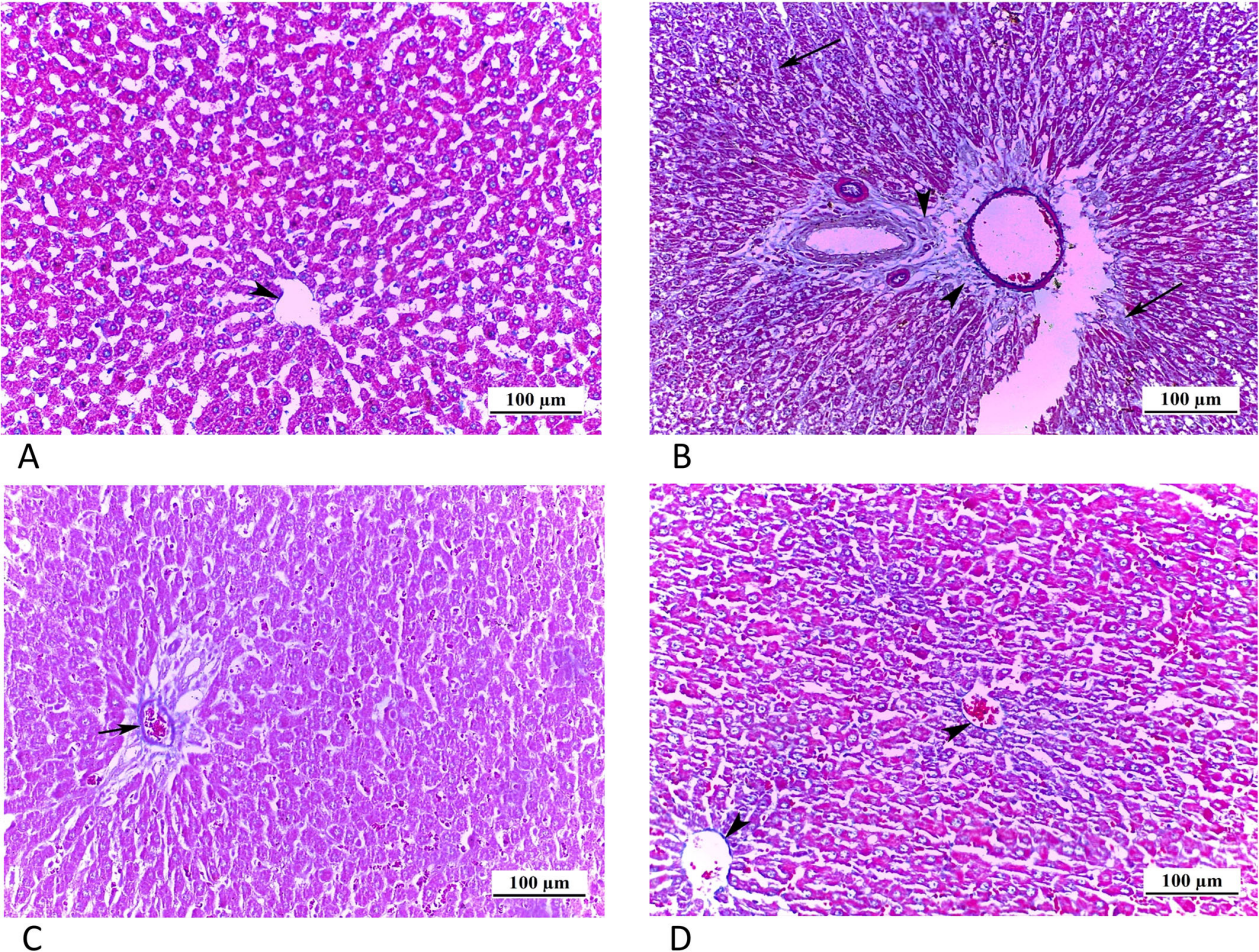
Photomicrograph of liver sections: **(A)** CTRL grp showing scanty collagen fibers (arrowhead). **(B)** Cd grp showing a substantial increment in collagen fibers in portal area (arrow heads) and between hepatic cords (arrows). **(C)** Cd + Cana grp showing few amounts of collagen deposition (arrow) in the portal area. **(D)** Cd + Sita grp showing some deposited collagen fibers (arrowheads) encompassing central vein. (Masson’s Trichrome ×200).

**Figure 8:**
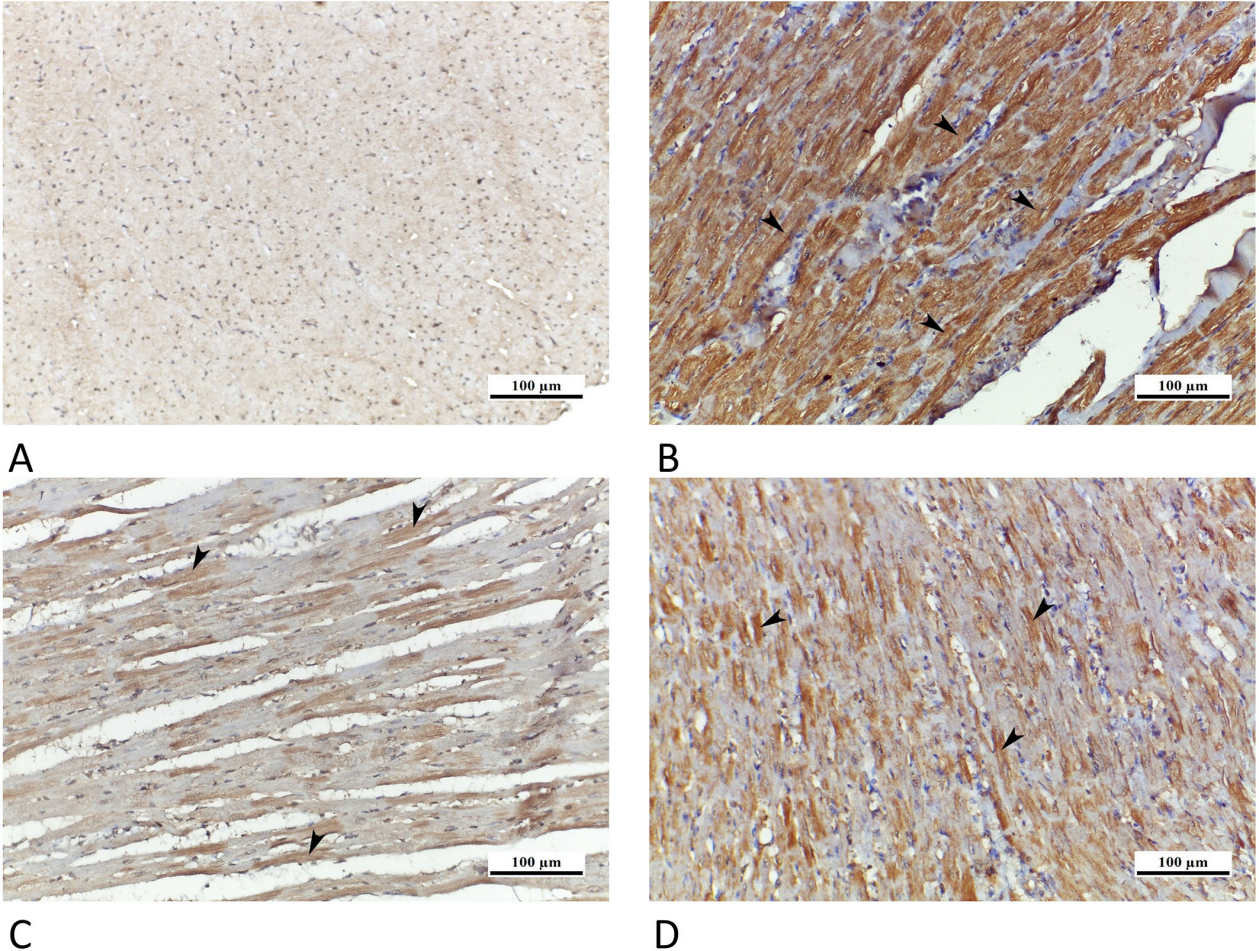
Photomicrographs of myocardial sections: **(A)** CTRL grp showing caspase 3 negative immunoreaction. **(B)** Cd grp showing caspase 3 highly positive immunoreaction (arrowheads). **(C)** Cd + Cana grp displaying caspase 3 mildly positive immunoreaction (arrowheads). **(D)** Cd + Sita grp revealing caspase 3 moderately positive immunoreaction in cardiomyocytes (arrowheads). (Caspase 3 × 200).

**Figure 9:**
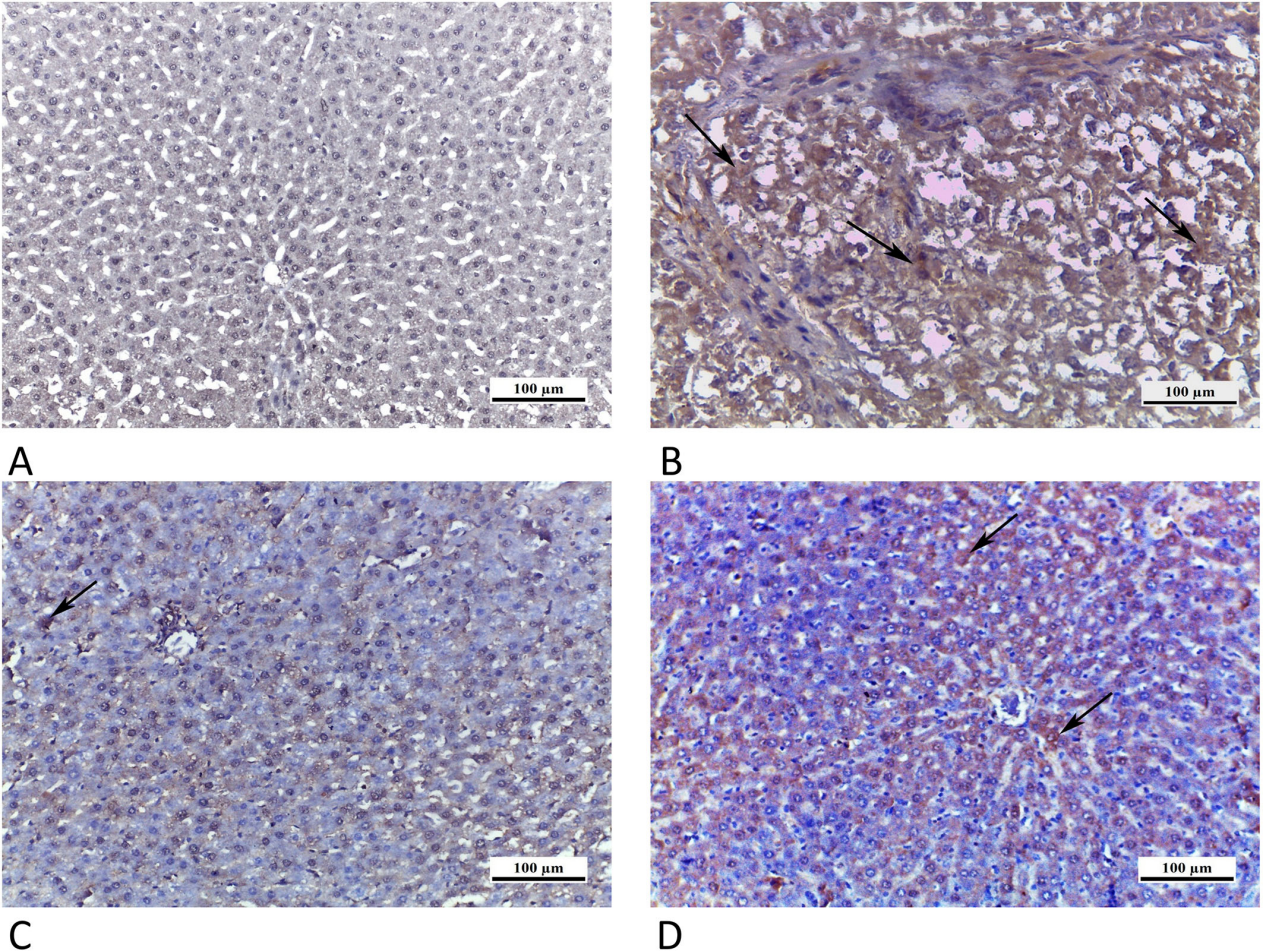
Photomicrographs of liver sections: **(A)** CTRL grp presenting a caspase 3 negative immunoreaction. **(B)** Cd grp showing enhanced caspase 3 cytoplasmic immunoreaction (arrows). **(C)** Cd + Cana grp showing mild positive cytoplasmic immunoreaction for caspase 3 (arrow). **(D)** Cd + Sita grp demonstrating caspase 3 moderately positive cytoplasmic immunoreaction (arrows). (Caspase 3 × 200).

**Figure 10:**
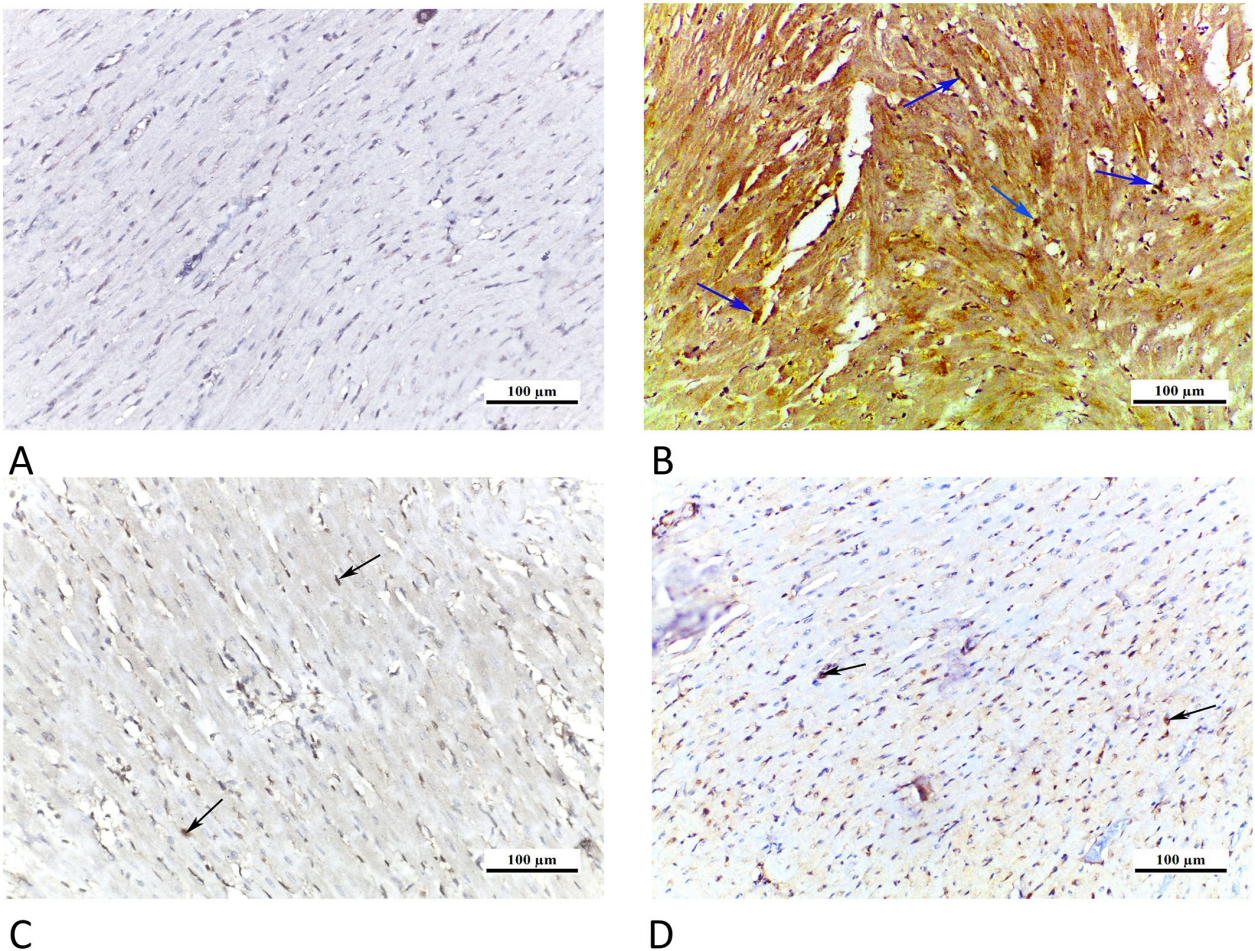
Photomicrographs of myocardial sections from different groups: **(A)** CTRL grp presenting a negative NF-κB immunoreaction. **(B)** Cd grp revealing high positive NF-κB nuclear/paranuclear immunoreaction (arrows). **(C)** Cd + Cana grp showing mild positive nuclear/paranuclear immunoreaction for NF-κB (arrow). **(D)** Cd + Sita grp exhibiting moderately positive NF-κB nuclear/paranuclear immunoreaction (arrows). (NF-κB ×200).

**Figure 11:**
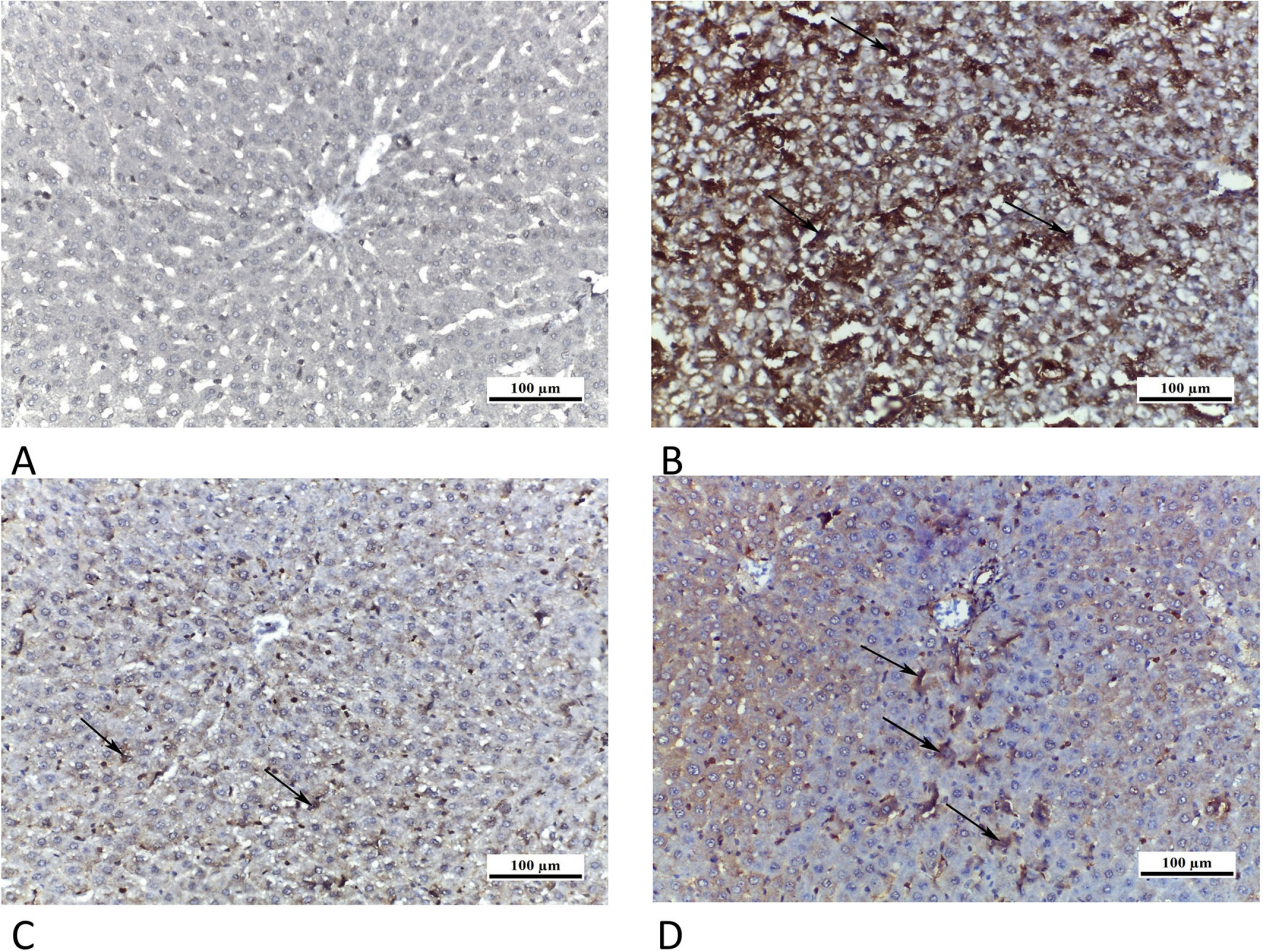
Photomicrographs of liver sections: **(A)** CTRL grp expressing a negative NF-κB immunoreaction. **(B)** Cd grp demonstrating a high positive NF-κB nuclear/paranuclear immunoreaction (arrows). **(C)** Cd + Cana grp showing mild positive nuclear/paranuclear immunoreaction for NF-κB (arrows). **(D)** Cd + Sita grp showing moderately positive nuclear/paranuclear NF-κB immunoreaction (arrows). (NF-κB ×200).

**Figure 12:**
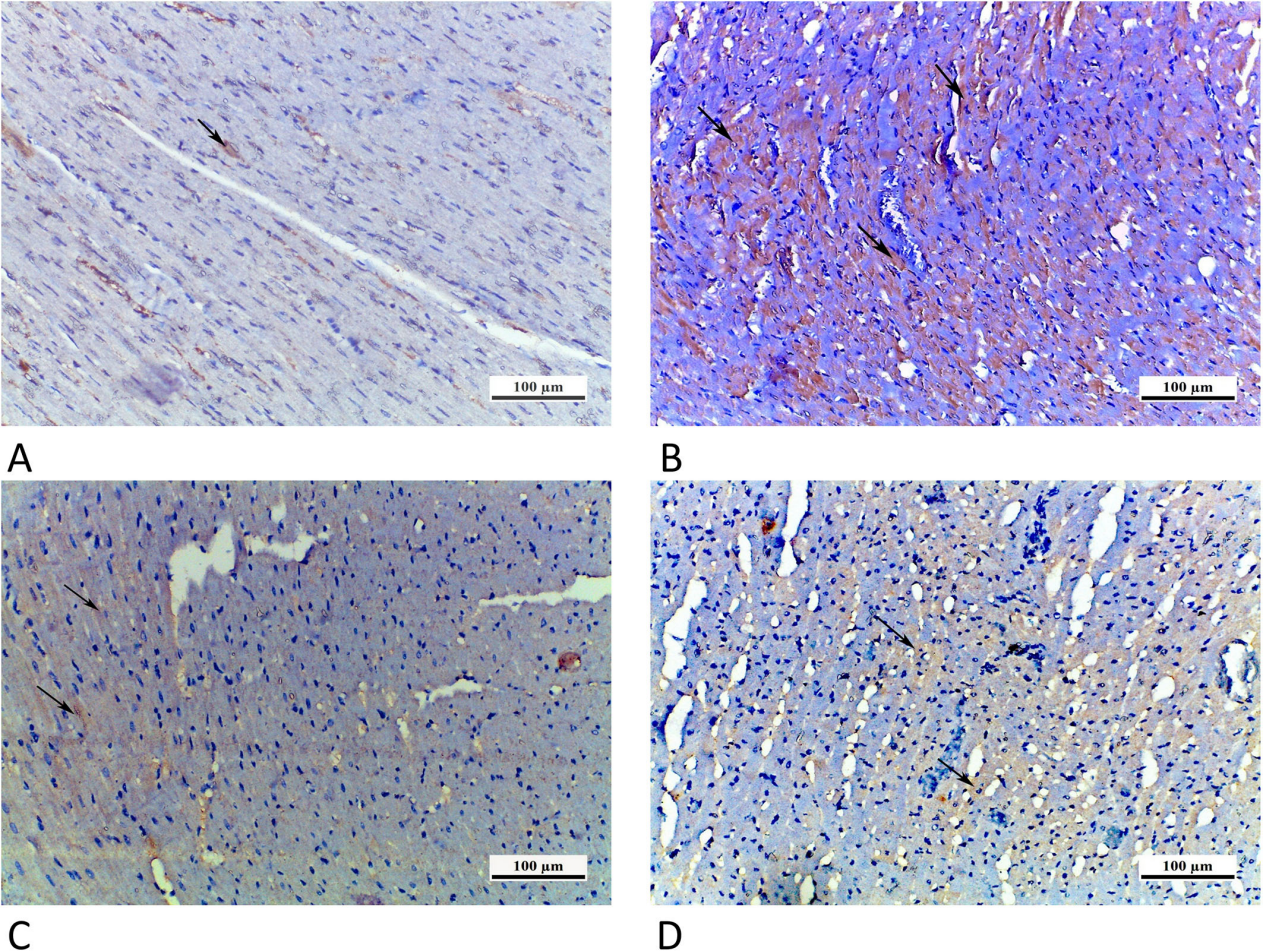
Photomicrographs of myocardial sections where cytoplasmic iNOS immunoreaction was weak (arrow) in CTRL grp **(A)**, highly positive (arrows) in Cd grp **(B)**, weakly positive (arrows) in Cd + Cana grp **(C)** and mildly positive (arrows) in Cd + Sita grp **(D)**. (iNOS ×200).

**Figure 13:**
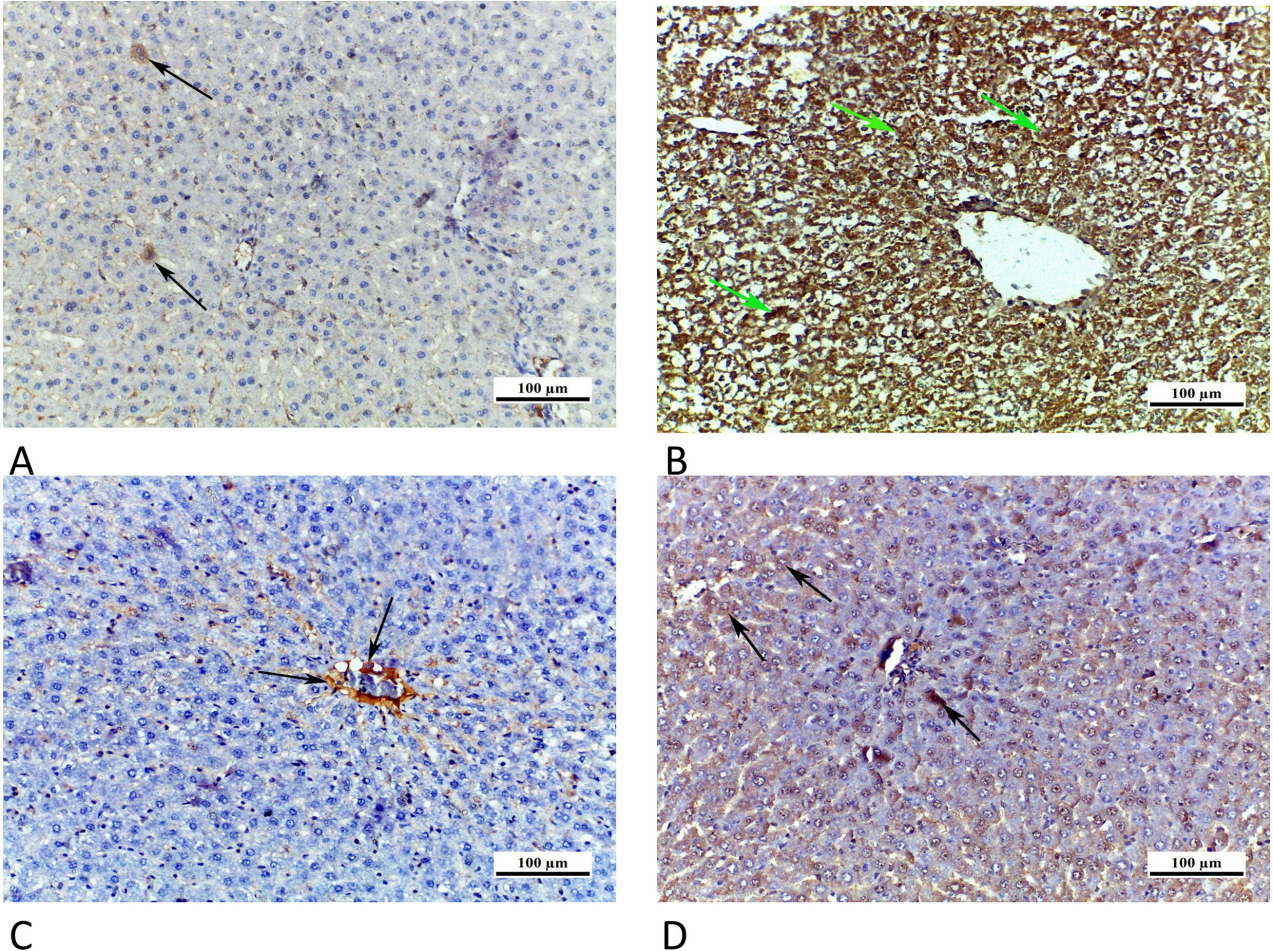
Photomicrographs of liver sections in which cytoplasmic iNOS immunoreaction (arrows) was weak in CTRL grp **(A)**, strongly positive in Cd grp **(B)**, mildly positive in Cd + Cana grp **(C)** and moderately positive in Cd + Sita grp **(D)**. (iNOS ×200).

The original version of this article has been updated.

